# IPA-ANN: A Novel Framework for Optimizing Artificial Neural Network Weights and Biases Using Immune Plasma Algorithm

**DOI:** 10.3390/biomimetics11080597

**Published:** 2026-08-20

**Authors:** Sercan Demirci, Durmuş Özkan Şahin, Gülcan Yıldız, Doğan Yıldız, Samad Hasanlı

**Affiliations:** 1 Department of Computer Engineering, Faculty of Engineering, Ondokuz Mayıs University, Atakum, Samsun 55139, Turkey; sercan.demirci@omu.edu.tr (S.D.); gulcan.ozer@omu.edu.tr (G.Y.); 2 Department of Electrical and Electronics Engineering, Faculty of Engineering, Ondokuz Mayıs University, Atakum, Samsun 55139, Turkey; dogan.yildiz@omu.edu.tr; 3 Department of Computer Engineering, Institute of Graduate Studies, Ondokuz Mayıs University, Atakum, Samsun 55139, Turkey; samad.hasanli@bil.omu.edu.tr

**Keywords:** metaheuristic algorithms, immune plasma algorithm, artificial neural network, classification, classification problems, weight optimization

## Abstract

Classification is a fundamental technique in data mining that predicts categorical labels by analyzing input features. However, training Artificial Neural Networks (ANNs) using traditional methods often encounters challenges, such as getting stuck in local minima and slow convergence. To address these issues, this study proposes a novel hybrid model, IPA-ANN, which integrates the Immune Plasma Algorithm (IPA) to optimize the ANN’s connection weights and biases. The IPA, inspired by the immune plasma treatment process, utilizes a unique donor-receiver mechanism to balance exploration and exploitation in the search space. The proposed model was evaluated on nine benchmark datasets from the UCI repository and compared with 18 state-of-the-art metaheuristic algorithms, including Grey Wolf Optimization (GWO), Differential Evolution (DE), and Particle Swarm Optimization (PSO). Experimental results were analyzed using accuracy, F1-score, confusion matrices, and convergence graphs. The findings indicate that IPA-ANN achieves competitive and stable classification performance across different datasets while demonstrating favorable convergence characteristics in several cases. Furthermore, the study investigates the influence of donor–receiver parameters on the optimization process, highlighting the adaptability of the proposed framework. The reliability of these findings was further examined through repeated stratified 5-fold cross-validation and paired Wilcoxon signed-rank tests with Holm–Bonferroni correction on representative datasets, confirming that a subset of the observed performance differences are statistically significant, and through a computational cost analysis showing that IPA-ANN incurs no additional overhead relative to the majority of the compared algorithms. This study contributes to the literature by presenting the first documented application of IPA in ANN training and by providing a modular infrastructure for future metaheuristic-based ANN optimization studies.

## 1. Introduction

Classification is a technique that enables the prediction of similar characteristics by examining categorical target or class variable values, and is used in the analysis of various types of data. Proper data management and the use of appropriate classification algorithms significantly facilitate information discovery. The classification technique predicts the outcome of a specific problem based on input features. Classification is mostly applied to classes defined as categories, targets, or labels. The main goal of the technique is to assign unknown patterns to the appropriate class. This technique is divided into four categories: binary classification, multi-class classification, imbalanced classification, and multi-label classification. Classification models have two learning strategies: lazy learners and eager learners. In lazy learners, the training data is used only when the test data is ready for classification. The algorithm does not create a model until it receives the test data. For example, the k-Nearest Neighbor (kNN) model works with lazy learning logic. In eager learners, the classification layer is created before the dataset is trained and tested. This type spends more time on dataset training and less time on prediction compared to lazy learning because it creates a classification layer. Decision Tree (DT), Naive Bayes (NB), and Artificial Neural Network (ANN) models work with this type of logic [1].

Metaheuristic algorithms play an important role in hyperparameter optimization for classification problems. Compared to manual tuning methods, these algorithms explore a wider search space, thereby improving classifier performance [2]. Gulmez’s study showed that ANN models optimized using metaheuristic approaches are successful in classification problems in the field of data mining. This approach particularly improves the generalization ability of classifiers in weight adjustment [3].

**Figure 1:**
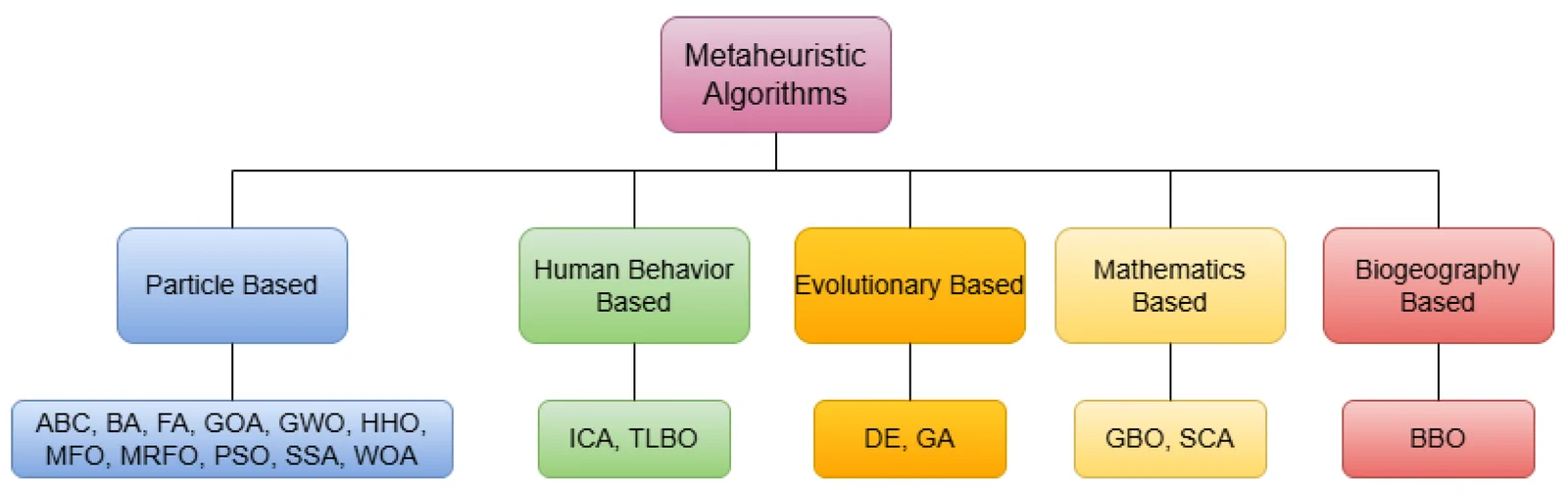
Compared metaheuristic algorithms.

There are many studies on the use of metaheuristic algorithms in conjunction with ANN. ANNs are considered active techniques for solving non-linear problems. However, an ANN may converge to a local minimum rather than a global minimum. This leads to the inappropriate use of a network structure during ANN training. To avoid these problems, various approaches, such as metaheuristic algorithms, have been integrated into the ANN model [4]. The process of optimizing the ANN model with metaheuristic algorithms typically follows similar steps, including the training error criterion, the generalization error criterion, and a penalty term related to network complexity. These are the criteria used in constructing the objective function [5]. Metaheuristic algorithms are used together to improve ANN convergence and increase robustness to data compression. Furthermore, metaheuristic algorithms have been shown to be highly effective for optimizing prediction models. While ANNs are effective at determining optimal weight and bias values, they may encounter optimization problems depending on the cost function’s characteristics [6]. Figure 1 shows a visual representation of the metaheuristic algorithms compared with the model developed in this study.

Yang introduced the Bat Algorithm (BA) to the literature. The algorithm is inspired by the echolocation behavior of bats [7]. Saba et al. developed a hybrid BA-ANN model to predict earthquakes. The BA algorithm was used to update the ANN weights. The developed model has been shown to achieve better accuracy than an ANN in predicting the magnitude and timing of possible earthquakes. The BA-ANN model, which uses a stochastic approach, tends to find global optima. The proposed model can be effectively used in applications that require prediction. This model is also useful in improving results because it can adjust and optimize parameters [8].

Yang introduced the Firefly Algorithm (FA), inspired by the light-emitting behavior of fireflies, and proposed it as a metaheuristic for multi-objective search and optimization problems [9]. In the study by Mostafaeipour et al., the FA and BA algorithms were combined to develop a hybrid approach for predicting air travel demand. Additionally, the ANN architecture was integrated into these metaheuristic algorithms. The results showed that the combined ANN improved the match with real data by up to 90%. This means the developed model performs better than other models [10].

In the study conducted by Mahadeva et al., an ANN model was proposed using the Grey Wolf Optimization (GWO) to optimize the permeate flow rate in a desalination plant. It was observed that the GWO-ANN model effectively optimized the network’s weight and bias parameters. It was also observed that the model reduced the mean-squared error and increased convergence speed during the learning phase. The proposed model was found to achieve better results compared to the other models [11].

Atashpaz-Gargari and Lucas introduced the Imperialist Competitive Algorithm (ICA), a new optimization algorithm inspired by imperialist competition [12]. Nosratabadi et al. compared the GWO-ANN and ICA-ANN models to predict crop yield. Several features were selected to evaluate the performance of the compared models. The most suitable feature sets were determined for each method. Based on the results obtained from the performance metrics, the GWO-ANN model was found to be superior in obtaining the best correlated models. According to the experimental results, the GWO-ANN model achieved better predictive performance [13].

Rojas developed the Backpropagation Algorithm (BPA) for use in the training of neural networks [14]. Yaghini et al. combined the BPA and PSO algorithms to optimize the weights of ANN prediction models. In this study, the proposed model was compared with three commonly used ANN training techniques in terms of accuracy and training time on eight benchmark problems. The comparisons were evaluated on card, cancer, diabetes, gene, heart, horse, iris, and thyroid datasets. The proposed model was compared with other methods for classification problems on these datasets and achieved the best classification performance. The evaluations showed that the proposed model yielded better results [15].

Isikdag et al. investigated the use of ANN architectures for estimating gas yield via the photocatalytic carbon dioxide reduction process. In the study, classification accuracy was analyzed using an ANN architecture with six metaheuristic algorithms on gas-phase and liquid-phase datasets. The analyses revealed an ANN architecture capable of achieving 94.61% classification accuracy in the gas-phase dataset and 90.79% in the liquid-phase dataset. When other classification techniques in the literature were examined, the best classification accuracy observed was 92% [16]. The values found were seen to be higher than those of traditional machine learning models. In line with the findings, it was seen that the use of ANNs greatly contributed to the estimation of the gas yield [17].

The COOT optimization algorithm was proposed by Naruei and Keynia [18]. Özden and İşeri used this algorithm in the training phase of the ANN [19]. In this study, the proposed COOT-ANN model was compared with Gradient Descent (GD)-, Scaled Conjugate Gradient (SCG)-, and Levenberg–Marquardt (LM)-based ANNs on four open-access classification datasets. Furthermore, Kayhan and İşeri demonstrated the application of the COOT algorithm to clustering problems [20]. In addition, the COOT algorithm is used to solve various other problems in the literature [21,22].

Mirjalili and Lewis developed the Whale Optimization Algorithm (WOA). This algorithm mimics the social behavior of humpback whales [23]. Haghnegahdar and Wang used the WOA-ANN model to classify attacks and failures in power systems with varying levels of difficulty. The WOA was used to obtain weights and deviations using mean-squared error for ANN training. Data preprocessing involved data cleaning. Evaluations such as accuracy, recall, precision, and F1-score were used for performance evaluation. The results showed that the proposed model successfully detected attack data [24]. In addition, the WOA algorithm is used to solve various other problems in the literature [25,26].

Although many metaheuristic-based ANN hybrid models have demonstrated promising results in classification and prediction problems, several important limitations still remain in the literature. Many existing approaches suffer from premature convergence, insufficient balance between exploration and exploitation, sensitivity to population diversity, or unstable performance across datasets with different structural characteristics. In addition, the optimization behavior of several widely used metaheuristics may strongly depend on specific search dynamics, which can limit their generalization capability in complex ANN weight optimization problems. Therefore, there is still a need for alternative metaheuristic frameworks capable of providing a more adaptive population interaction mechanism and a more balanced search strategy during ANN training. Motivated by these limitations, the present study investigates the potential of the Immune Plasma Algorithm (IPA), whose donor–receiver interaction structure offers a distinctive optimization mechanism for improving ANN weight and bias optimization.

The IPA is a method developed based on the plasma treatment process in the immune system and is recognized in the literature as a new metaheuristic [27]. IPA aims to establish a balanced optimization structure by maintaining diversity and convergence through donor–receiver population interactions. Unlike many conventional population-based optimizers, IPA incorporates a treatment-inspired adaptive interaction mechanism that may enhance search diversity and reduce premature convergence during optimization. However, despite these promising characteristics, there are currently no studies investigating the use of IPA for ANN weight and bias optimization in classification problems.

Therefore, this study demonstrates that the IPA can achieve successful results in optimizing ANN weights. In the study, IPA was compared with 18 metaheuristic algorithms across 9 datasets. Thus, the classification success and convergence performance of IPA were examined in detail with various performance metrics. While the primary objective of this work is to comparatively investigate the behavior and optimization capabilities of derivative-free, population-based metaheuristic approaches, a conventional gradient-based ANN baseline (trained with SGD, SGD-Momentum, RMSprop, Adam, and Adagrad) was additionally evaluated on two representative datasets (Iris and Chess) under an identical architecture and data partitioning scheme, in order to contextualize the practical benefit of the proposed framework relative to standard training methods.

The main contributions of this study to the literature can be summarized as follows:

- Application of a new metaheuristic algorithm to ANN: The study demonstrates the first use of IPA to optimize ANN weights and examines its effectiveness in improving the model’s classification accuracy. The global search features included in IPA have been applied to classification problems.
- Comprehensive comparative analysis: IPA was fairly evaluated under the same conditions with 18 different metaheuristic algorithms on 9 datasets. Performance was evaluated using metrics such as accuracy, F1-score, and a confusion matrix. This proved IPA’s generalization ability and success in optimization problems.
- High-performance results: The findings revealed that IPA achieved competitive or superior test accuracy relative to most metaheuristic algorithms across the majority of the datasets, an observation further supported by statistical significance testing conducted on representative datasets. In particular, the IPA-ANN model developed on medical datasets improved classification performance and convergence.
- Reusable modular infrastructure: This infrastructure, developed for the use of IPA in ANN optimization, provides a modular foundation that allows different metaheuristic algorithms to be compared within the same structure. Therefore, an infrastructure that can be used in optimization problems has been established.

## 2. Background Classification Optimization Formulation

This section outlines a general classification optimization framework commonly used in metaheuristic-based approaches. While the presented equations describe a simplified linear model, the proposed IPA-ANN method employs a nonlinear ANN architecture optimized using the cross-entropy loss function detailed in Section 3.3. In representing an X matrix of size MxD, a dataset containing 70 M different examples, each with D features, is used. To determine whether the sample labels or class in the X matrix are correctly predicted, the weight vector W = $\left\{w_{1},w_{2},\ldots ,w_{D}\right\}$ must satisfy Equation (1) [28,29,30].

(1) $$X\times W^{T}=Y\;\mathrm{or}\;\left[\begin{array}{cccc} x_{11}, & x_{12}, & \cdots , & x_{1D}\\ x_{21}, & x_{22}, & \cdots , & x_{2D}\\ \vdots & \vdots & \vdots & \vdots \\ x_{M1}, & x_{M2}, & \cdots , & x_{MD} \end{array}\right]\;\times \;\left[\begin{array}{c} w_{1}\\ w_{2}\\ \vdots \\ w_{D} \end{array}\right]=\left[\begin{array}{c} y_{1}\\ y_{2}\\ \vdots \\ y_{M} \end{array}\right]$$

A solution exists when R(X) = R(X, Y) < D. Here, R(X) is the rank of the X matrix, and R(X, Y) is the rank of the matrix formed by the combination of X and Y. Often, finding an exact solution can be difficult because the number of features is less than the number of examples [28]. Xue et al. stated that the classification output of an example can be updated by calculating the features and applying a prediction-based solution to overcome this difficulty. This is detailed in Equation (2) [28,29,30].

(2) A×WT≈Yorw1x11+w2x12+⋯+wDx1D≈y1w1x21+w2x22+⋯+wDx2D≈y2⋯w1xM1+w2xM2+⋯+wDxMD≈yM

Upon detailed examination of the classification problem definition, it is evident that the definition is transformed into a numerical optimization problem with an objective function to be minimized, as shown in Equation (3). Furthermore, the solution capabilities of metaheuristic algorithms have been tested in classification optimization [28,29,30].

(3) $$f=\sqrt{\sum_{i=1}^{M}\left(\sum_{j=1}^{D}{\left(w_{j}x_{ij}-y_{i}\right)}^{2}\right)}$$

Metaheuristic algorithms produce a weight vector (W) or a final solution based on minimizing the objective function. The correctness of the weight vector and the final solution is verified by checking whether the classes of the examples in X are correctly determined. By sequentially selecting i from the set {1,2,…,M} and defining it with a threshold value defined by δ, it is possible to decide how the part $w_{1}x_{i1}+w_{2}x_{i2}+\cdots +w_{D}x_{iD}$ approaches $y_{i}$. Examining Equation (4), if the result of $w_{1}x_{i1}+w_{2}x_{i2}+\cdots +w_{D}x_{iD}-y_{1}$ is equal to or greater than −δ and less than δ, the label of the $x_{i}$ example is considered to be correctly determined [30,31].

(4) −δ≤w1x11+w2x12+⋯+wDx1D−y1<δ−δ≤w1x21+w2x22+⋯+wDx2D−y2<δ⋯−δ≤w1xM1+w2xM2+⋯+wDxMD−yM<δ

Another aspect to consider in classification optimization is how the lower bound (lb) and upper bound (ub) of the elements in the weight vector are determined. The function in Equation (5) is presented for determining the lower and upper bounds. In this equation, σ is a constant that adjusts the distance between the lower and upper bounds. N represents the number of examples in the dataset [30,31].

(5) $$\left(\mathrm{ub},\mathrm{lb}\right)=\pm \sigma \frac{\sum_{i=1}^{N}y_{i}}{\sum_{i=1}^{N}\sum_{j=1}^{D}x_{ij}}$$

While the above formulation outlines a general classification optimization framework, the proposed IPA-ANN model employs a nonlinear ANN structure optimized using a cross-entropy-based fitness function rather than the squared-error approach presented here.

## 3. Proposed Hybrid Model: IPA-ANN

This section explains the stages and working principle of the IPA. It provides basic information about the general ANN structure. Furthermore, the features and flowchart of the developed IPA-ANN model are explained in detail.

### 3.1. Immune Plasma Algorithm

Following the COVID-19 pandemic, a treatment method known as the immune plasma method began to be implemented worldwide. The fundamental idea behind the immune plasma method is to treat critically ill individuals using the antibody-rich portion of blood obtained from individuals who had contracted the disease and recovered.

Aslan and Demirci developed the immune plasma method and introduced a new metaheuristic algorithm, IPA. In IPA, each individual in the population is matched with a possible solution to the optimization problem to be solved, and this individual’s immune response to infection is represented as fitness. The basic steps of IPA are explained in detail below [27].

#### 3.1.1. Generating Initial Individuals

Individuals in the IPA represent a possible solution to the problem being addressed. In an optimization problem with D different parameters, the kth individual $x_{k}$ in the population size (PS) can be generated using Equation (6). In this equation, $x_{kj}$ is matched with the jth parameter of the $x_{k}$ individual. Additionally, $x_{j}^{\mathrm{low}}$ and $x_{j}^{\mathrm{high}}$ represent the lower and upper bounds of the jth parameter. rand(0,1) selects a random value between 0 and 1 [32].

(6) $$\begin{array}{cc} \; & x_{kj}=x_{j}^{\mathrm{low}}+\mathrm{rand}\left(0,1\right)\cdot \left(x_{j}^{\mathrm{high}}-x_{j}^{\mathrm{low}}\right)\\ \; & k\in \{1,2,\ldots ,PS\},\;j\in \{1,2,\ldots ,D\} \end{array}$$

#### 3.1.2. Infection Spreading and Immune System Response

A small proportion of infectious individuals carrying antigens can affect the entire population. When the secretions of an infectious individual interact with another individual’s body, the host mounts a specific response to protect itself against the secretion. Equation (7) provided by IPA is used to model how an individual affects another individual and triggers the immune system to create a specific response. In Equation (7), $x_{kj}$ represents the randomly determined jth parameter of the infected individual $x_{k}$, while $x_{kj}^{\inf}$ represents the jth parameter to be recalculated for the infected $x_{k}$. Finally, $x_{mj}$ is the jth parameter of the previously infected and randomly selected individual $x_{m}$ [32].

(7) $$\begin{array}{cc} \; & x_{kj}^{\inf}=x_{kj}+\mathrm{rand}\left(-1,+1\right)\cdot \left(x_{kj}-x_{mj}\right)\\ \; & k\in \{1,2,\ldots ,PS\},\;m\in \{1,2,\ldots ,PS\}-\{k\} \end{array}$$

If the immune system of individual $x_{k}$ responds and specific antibody production increases, this indicates that the immune system has recognized the antigens or updated its immune memory against the mutants. In the algorithm, there is a direct relationship between the individual’s specific antibody amount and the value in the objective function. Considering that F is the objective function and $x_{k}$ is the kth individual in the PS, when the objective function value denoted by $x_{k}^{\inf}$ and f($x_{k}^{\inf}$) is better than the objective function value of $x_{k}$ denoted by f ($x_{k}$), we see that the jth parameter of $x_{k}$ is set to $x_{k}^{\inf}$. Otherwise, the jth parameter of the $x_{k}$ individual remains as in Equation (8) from the minimization problem perspective [32].

(8) $$x_{kj}=\left\{\begin{array}{cc} x_{kj}^{\inf}; & \mathrm{if}\;f\left(x_{kj}^{\inf}\right)<f\left(x_{k}\right)\\ x_{kj}; & \mathrm{if}\;f\left(x_{kj}^{\inf}\right)\geq f\left(x_{k}\right) \end{array}\right.$$

#### 3.1.3. Plasma Extraction and Transfer

Individuals’ immune system responses to infections vary. Some infected individuals require care, while others recover quickly. Individuals who recover quickly contribute to the treatment process. The convalescent plasma, or immune plasma, method is one of the ways that contribute to individuals’ recovery. In the algorithm, the number of donors (NoDs) and the number of receivers (NoRs) are determined before the plasma transfer process begins. Once these parameters are provided, IPA determines which individuals will serve as donors and which will serve as recipients. Receiver individuals are identified as the NoR individuals with the lowest performance, while donor individuals are identified as the NoD individuals with the best performance in the PS size. IPA uses Equation (9) to model the plasma transfer from the $x_{m}^{\mathrm{dnr}}$ individual to the $x_{k}^{\mathrm{rcv}}$ individual. In the equation, $x_{k}^{\mathrm{rcv}}$ is the kth receiver individual, and $x_{m}^{\mathrm{dnr}}$ is a randomly selected donor individual [32].

(9) $$\begin{array}{cc} \; & x_{kj}^{\mathrm{rcv}\text{-}p}=x_{kj}^{\mathrm{rcv}}+\mathrm{rand}\left(-1,+1\right)\cdot \left(x_{kj}^{\mathrm{rcv}}-x_{mj}^{\mathrm{dnr}}\right)\\ \; & j\in \{1,2,\ldots ,D\} \end{array}$$

Here, $x_{k}^{\mathrm{rcv}\text{-}p}$ represents the individual’s $x_{k}^{\mathrm{rcv}}$ after plasma therapy; $x_{kj}^{\mathrm{rcv}\text{-}p}$ represents the value of $x_{k}^{\mathrm{rcv}\text{-}p}$ associated with the individual’s jth parameter. If it contributes to $x_{k}^{\mathrm{rcv}}$ when the first dose is administered and the immune response is better than that of the individual with $x_{m}^{\mathrm{dnr}}$, it is assumed that the receiver of $x_{k}^{\mathrm{rcv}}$ can resist infection just like the donor of $x_{m}^{\mathrm{dnr}}$.

#### 3.1.4. Donor Update

After the plasma phase is complete, IPA applies a controlled-randomized approach to replace pre-selected donor individuals. If the random number generated between 0 and 1 is not greater than the ratio between the current fitness evaluation ($t_{cr}$) and the maximum fitness evaluation ($t_{max}$), each parameter of the $x_{m}^{\mathrm{dnr}}$ individual is changed according to the pre-assigned values. This change is detailed in Equation (10) [32].

Subsequent evaluations observed an increased probability of preserving the antibody property of the donor individual. If the random number generated between 0 and 1 is greater than the value $t_{cr}/t_{max}$, the individual’s immune system response is considered strong but insufficient to form antibody memory. In this case, the individual’s parameters are restarted using Equation (6) [32].

(10) $$x_{mj}^{\mathrm{dnr}}=x_{mj}^{\mathrm{dnr}}+\mathrm{rand}\left(-1,1\right)\cdot x_{mj}^{\mathrm{dnr}}$$

**Figure 2:**
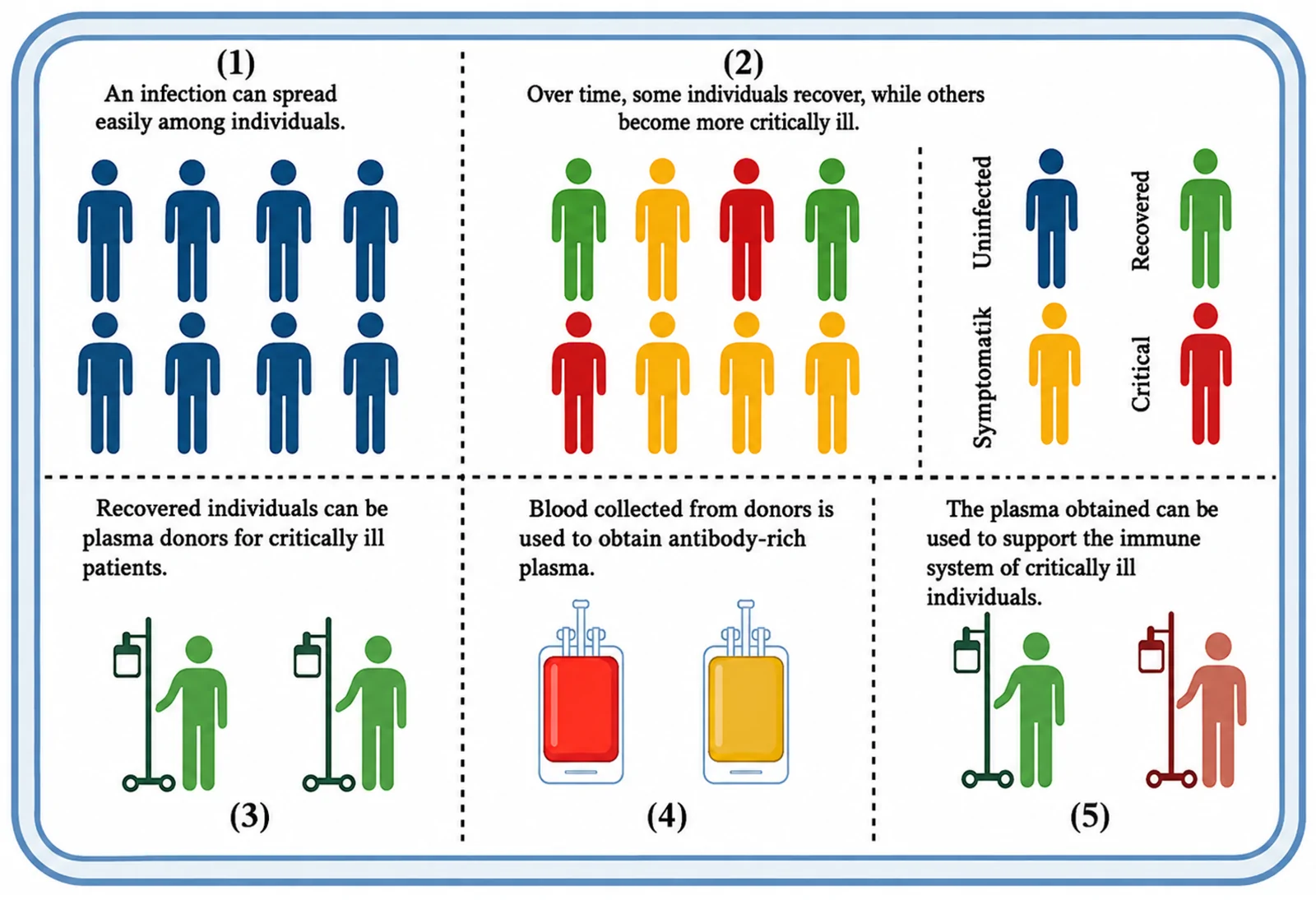
General concept of the IPA [27].

The general concept of IPA is shown in Figure 2. While some infected individuals recover, others become critically ill. Recovered individuals can become plasma donors for those in critical condition. The blood obtained is used to support the immune systems of critically ill individuals. The pseudocode for IPA is explained in detail in Algorithm 1.

### 3.2. Artificial Neural Network

An ANN is a mathematical model that simulates the functions and structures found in neural networks. The basic structure of every ANN model consists of neurons and has three sets of rules: multiplication, addition, and activation. In the input section, inputs are weighted, meaning values are multiplied by separate weights. In the middle section, the inputs and the error are subjected to an addition operation. In the output section, the activation function is applied after the error is summed [33].

The ANN structure consists of three layers: input, hidden, and output. The input layer is where external inputs are received. The hidden layer establishes the connection between inputs and outputs and uses synaptic weights. The output layer propagates the problem outputs [34]. ANNs have four characteristics: nonlinear, non-qualitative, non-convex, and unbounded. ANNs are composed of nodes, which serve as the structure’s memory. The weights used represent the connection between two nodes. The processing unit of the architecture consists of basic components representing different data, such as letters, objects, and features [35].

Recently, the use of metaheuristic algorithms for ANN optimization has increased [36,37]. These algorithms are used to optimize ANN parameters and structures. In the literature, researchers train ANN hyperparameters by combining metaheuristic algorithms [38,39]. The hybrid algorithms created solve the ANN optimization problem and also improve its performance. Finally, the use of metaheuristics helps increase accuracy, improve the learning process, and reduce execution time [40].
**Algorithm 1:** IPA pseudocode
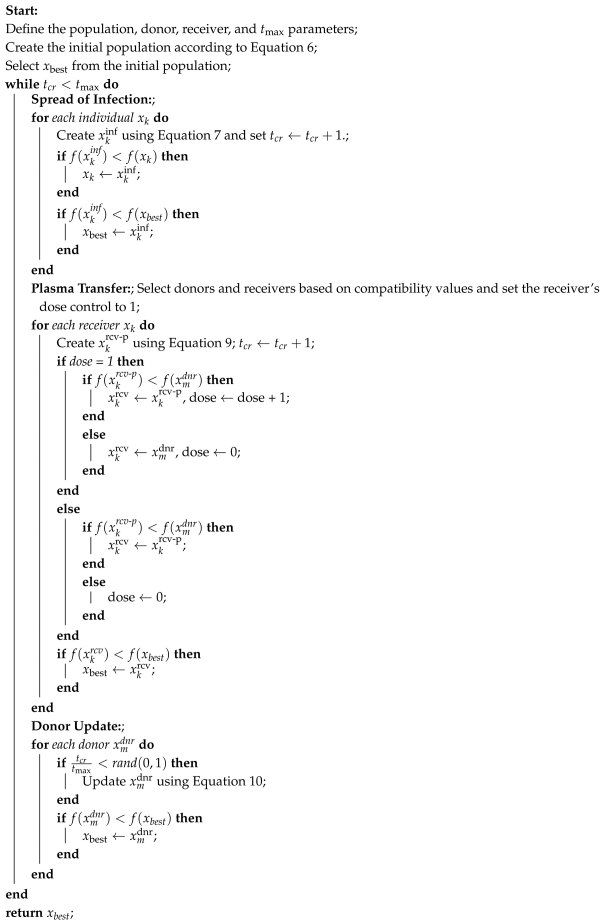


### 3.3. IPA-ANN

In this study, IPA was used to optimize the ANN’s weights and biases. Solutions in fitness evaluation evolve through mechanisms in the immune system and interact with one another to improve population fitness.

In the proposed model, the initial population is created with a population consisting of N individuals, each of D dimensions. For the current individual $x_{k}$, a random $x_{m}$ is selected from the population. The cross-entropy loss is calculated for each $x_{k}$ as the fitness function, and the best individual is selected. In contrast to the simplified squared-error formulation presented in Section 2, the proposed IPA-ANN framework employs categorical cross-entropy as the fitness function, as it is better suited for probabilistic ANN-based multi-class classification and enables more stable optimization. The population is sorted from smallest to largest according to the f($x_{k}$) value, and the first individual is taken to the donor pool $x_{m}^{\mathrm{dnr}}$. The remaining individuals go to the receiver pool $x_{k}^{\mathrm{rcv}}$. A j-dimensional random donor is selected from $x_{m}^{\mathrm{dnr}}$. Subsequently, a j-dimensional donor and candidate index are selected. The fitness function is recalculated for $x_{k}$ in the updated individuals. The f($x_{k}$) with the lowest value is selected as the best solution.

**Figure 3:**
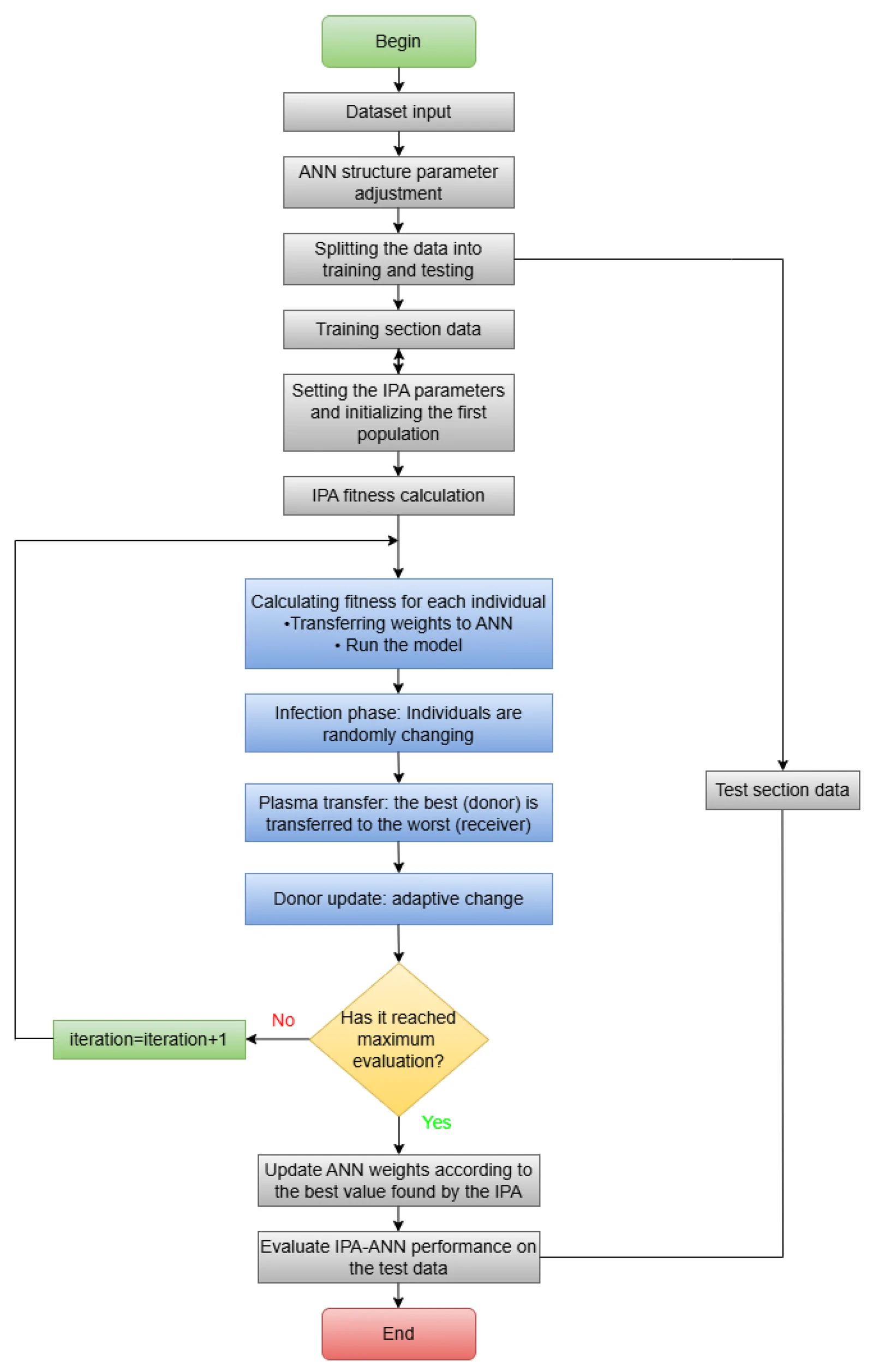
Flowchart of the IPA-ANN model.

In the proposed model, issues in the datasets were first resolved, and the datasets were defined. Subsequently, the ANN structure was defined. This structure consists of two hidden layers: one with 10 nodes and the other with 8. The input layer value is determined by the number of features in the dataset, while the number of classes determines the output layer value. The data in the defined datasets were divided into training and test sets. The IPA parameters were set, and the population was initialized. After the population started, fitness was evaluated. Then, plasma and antibody individuals were selected. In this part, antibodies were updated based on the plasma in which they were present. The fitness values of the updated antibodies were recalculated, and the old population was updated with the new individuals. After the best solution is found, if the maximum evaluation is reached, the ANN weights and the local best solution are updated. Finally, the IPA-ANN performance is evaluated on the test data. Figure 3 shows the flowchart stages of the proposed model.

**Figure 4:**
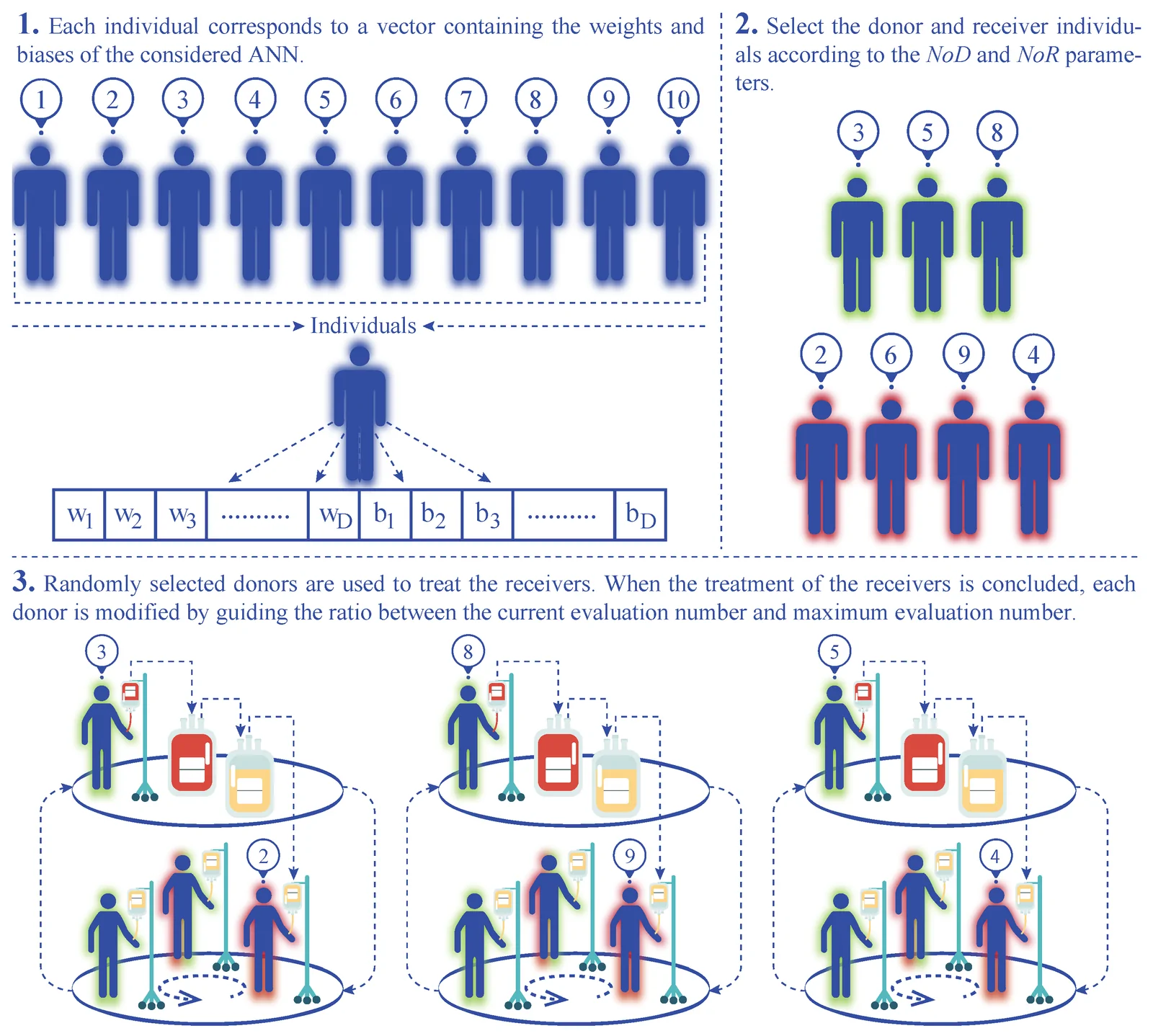
General architecture of the proposed IPA-ANN model.

The overall architecture and operational logic of the proposed IPA-ANN model are visualized in Figure 4. According to this architecture, each individual in the population represents a solution vector comprising weight and deviation values to be optimized for the ANN model. During the process, individuals are grouped as donors and recipients based on their performance under the NoD and NoR parameters. As detailed in Figure 4, randomly selected donor individuals are used to “treat” (improve solutions) recipients, and when the treatment process is complete, donors are updated with an adaptive change guided by the ratio of the current number of assessments to the maximum number of assessments. This integrated structure allows IPA to optimally determine the ANN’s connection parameters and improve its classification performance by utilizing the plasma transfer mechanism in the immune system.

In the proposed model, the ANN architecture is implemented as a fully connected feedforward network. The two hidden layers, consisting of 10 and 8 neurons, respectively, employ the Rectified Linear Unit (ReLU) activation function, which introduces non-linearity while mitigating the vanishing gradient problem. The output layer utilizes the Softmax activation function, which converts the raw output scores into a normalized probability distribution across the target classes; this formulation is applied uniformly to both binary and multi-class classification problems considered in this study. The final class prediction for a given sample is obtained by selecting the class index corresponding to the maximum probability value in the Softmax output vector (argmax decision rule). Class labels were encoded using one-hot encoding, which is compatible with the Softmax output layer and the cross-entropy-based fitness function. All fully connected layers, including both hidden layers and the output layer, incorporate bias terms in addition to their respective weight matrices. Accordingly, the dimensionality (D) of the search space optimized by IPA corresponds to the total number of trainable parameters in the network, namely the flattened concatenation of the weight matrices and bias vectors of all three layers (W1, b1, W2, b2, W3, b3). During the optimization process, each candidate solution vector generated by IPA is reshaped into the corresponding weight and bias tensors prior to being assigned to the ANN for fitness evaluation.

This architecture was deliberately kept fixed across all nine datasets in order to isolate the effect of the optimization algorithm on classification performance, rather than introducing dataset-specific architecture tuning as an additional confounding factor across the 19 compared algorithm configurations. The selected hidden-layer sizes follow common practice in prior metaheuristic–ANN hybridization studies, providing a balance between sufficient representational capacity and a computationally tractable weight/bias search space across datasets with varying input dimensionality. Adapting the network architecture to the complexity and dimensionality of each dataset is identified as a direction for future work.

## 4. Experimental Settings

This section provides a detailed explanation of the datasets used to evaluate the proposed model against different metaheuristic algorithms. The data preprocessing step is also explained. The performance metrics used to evaluate the classification performance of the compared algorithms are explained in detail. Finally, the parameter adjustments for the metaheuristics are also explained.

### 4.1. Used Datasets

The datasets used in the study’s practical application were obtained from the University of California, Irvine (UCI) Machine Learning Repository [41]. The selected datasets were chosen by evaluating the most commonly used datasets in classification problems in the literature [42,43].

The selected datasets consist of various patterns used in classification problems. They also contain various features, examples, and classes required in practical applications. Table 1 lists the number of features, examples, classes, and class labels for the datasets. Table 2 contains the abbreviations of the datasets used in the study.

### 4.2. Data Preprocessing

**Figure 5:**
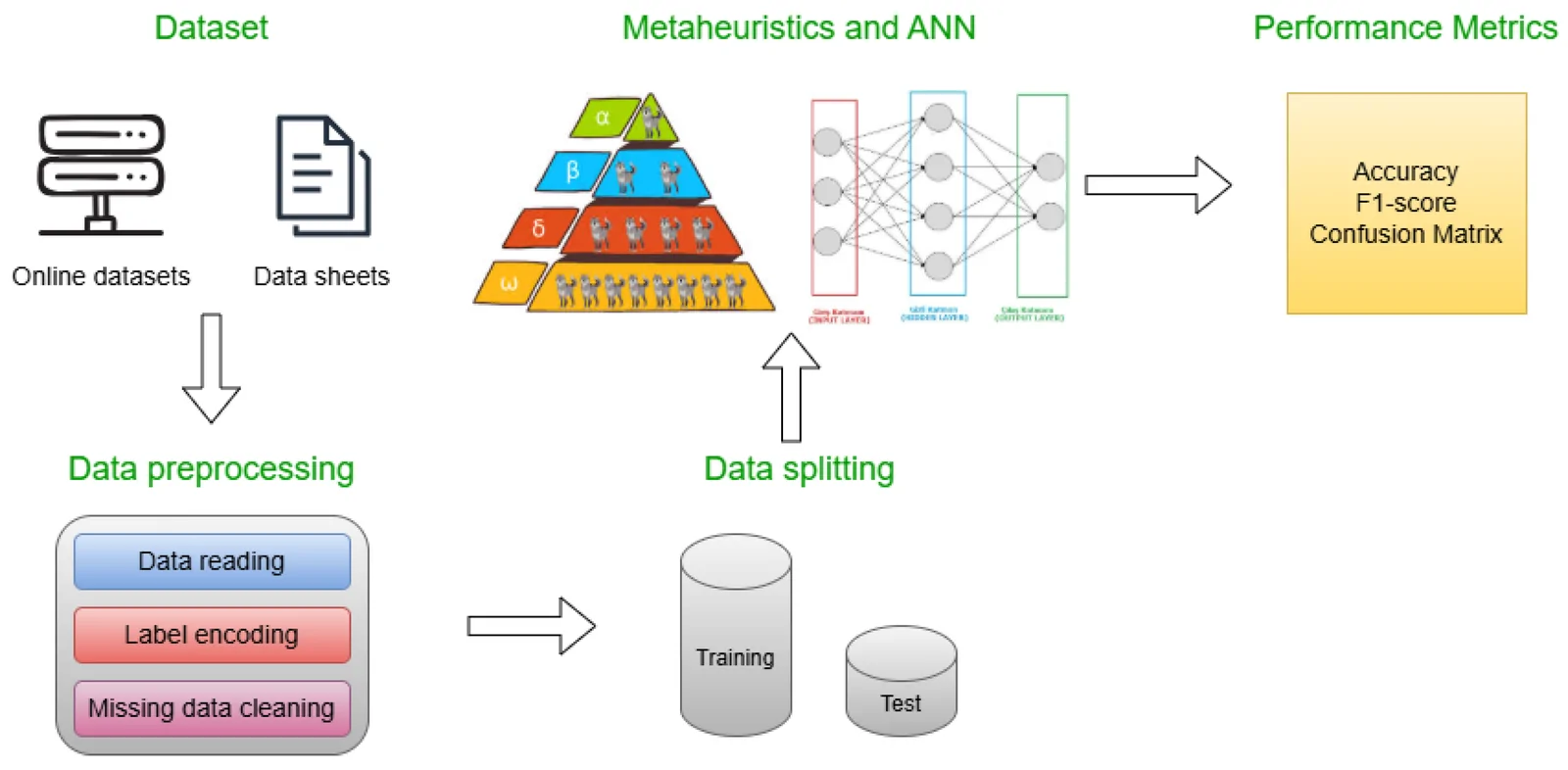
Process flowchart for the proposed IPA-ANN model.

Data preprocessing was performed on the datasets used in the study. Missing values in the Bare_nuclei feature in DS1 were converted to numerical form after being defined as missing values (NaN) using “?” symbols, and then filled with the mean of the column. All categorical features in DS2 were converted to numerical form using the label encoding method. In DS3, “?” symbols were defined as NaN. Missing values for categorical variables were imputed using the mode, while those for numerical variables were imputed using the mean value. Categorical features were converted to numerical form using label encoding. In DS4, categorical features were converted into numerical form using the label encoding method. The “?” symbols in the specified features in DS5 were defined as NaN, which were then converted to a numerical type and filled with the mean of the relevant features. Missing values in DS6 were marked as NaN, and missing values in numerical features were filled with the mean value. Only data reading was performed on the DS7. In DS8, categorical variables were converted into numerical form using the label encoding method. In DS9, “?” symbols were defined as missing values (NaN); missing values in categorical features were imputed using the mode of the relevant features. Subsequently, categorical features were converted to numerical form using the label encoding method. Figure 5 shows the process flowchart used in the study in detail.

To prevent data leakage between the training and test partitions, all preprocessing operations that involve statistical estimation from the data were fitted strictly on the training subset. Specifically, missing-value imputation statistics (mean values for numerical features and mode values for categorical features), label encoding mappings, and Min-Max normalization parameters (minimum and maximum values) were computed exclusively from the training data. The resulting transformations were subsequently applied to both the training and test subsets without recomputing any statistics from the test data, ensuring that no information from the test set influenced the preprocessing pipeline. Regarding feature scaling, Min-Max normalization to the [0, 1] range was applied to datasets exhibiting heterogeneous feature magnitudes (DS3, DS4, DS5, and DS6), since both ANN training and the IPA-based weight search are sensitive to feature scale. For datasets whose features were already on comparable or bounded numerical ranges (DS7 and DS9), normalization was not applied, as it was not expected to have a substantial effect on model performance.

### 4.3. Evaluation Metrics

Performance evaluation metrics are used to assess model performance in classification problems, and these values are typically calculated based on the confusion matrix. The values listed below are generally used as basic metrics [44].

- True Positive (TP): The number of positive data correctly predicted by the model.
- True Negative (TN): The number of negative data correctly predicted by the model.
- False Positive (FP): The number of positive data incorrectly predicted by the model.
- False Negative (FN): The number of negative data incorrectly predicted by the model.

Accuracy is the rate at which the data used in training the model correctly classifies the data in the test set. It is expressed as the ratio of the number of data points correctly predicted by the model to the total number of data points [44]. The formula for accuracy evaluation is shown in Equation (11).

(11) $$\mathrm{Accuracy}=\frac{TP+TN}{TP+TN+FP+FN}$$

The confusion matrix is a commonly used performance metric for evaluating classification models [45]. The matrix shows the relationship between the classifier’s predicted classes and the actual classes. Generally, each row corresponds to the actual class, and each column corresponds to the predicted class.

The confusion matrix usually contains four key elements: TP, TN, FP, and FN. These values can be used to calculate performance metrics such as sensitivity, specificity, accuracy, and F1-score [46]. These metrics play a critical role in accurately analyzing classifier performance on datasets with imbalanced class distributions. The confusion matrix can be applied to both binary and multi-class classification problems and reveals in detail which classes the model is more or less successful in [45].

The F1-score is obtained from the harmonic mean of the sensitivity and precision metrics. Sensitivity indicates how many true positives are correctly predicted. Precision indicates how many of the data predicted as positive are actually positive. Since sensitivity and precision alone are insufficient for performance evaluation, the F1-score metric was developed by combining the two metrics for a more efficient performance assessment [44]. Equations (12)–(14) show the sensitivity, precision, and F1-score functions, respectively.

(12) $$\mathrm{Sensitivity}=\frac{TP}{TP+FN}$$

(13) $$\mathrm{Precision}=\frac{TP}{TP+FP}$$

(14) $$F1\text{-}\mathrm{score}=2\times \frac{\mathrm{Precision}\times \mathrm{Sensitivity}}{\mathrm{Precision}+\mathrm{Sensitivity}}$$

### 4.4. Metaheuristic Parameter Adjustments

To ensure a fair and unbiased comparison among the 18 competing metaheuristic algorithms and the proposed IPA-ANN model, all algorithms were evaluated under identical experimental conditions, namely the same population size (PS = 30) and the same maximum evaluation count (10,000), across all nine datasets. Rather than performing per-dataset parameter tuning for each algorithm, the algorithm-specific parameters were fixed to the values most commonly recommended in the corresponding original proposing studies and widely adopted in the comparative metaheuristic optimization literature. This choice was made deliberately: subjecting each of the 19 compared algorithms to independent per-dataset tuning would introduce an additional, uncontrolled source of variability, as differences in the tuning budget or tuning strategy could disproportionately benefit certain algorithms and thereby confound the comparison. By instead adopting literature-consistent default configurations under a shared population size and evaluation budget, the comparison isolates and reflects the inherent search and convergence characteristics of each algorithm rather than differences attributable to tuning effort, which is consistent with common practice in the metaheuristic benchmarking literature.

The common parameters of the metaheuristic algorithms used in the study are set to PS 30, with a maximum evaluation count of 10,000. In the original parameters section, the following parameters were set: for Artificial Bee Colony (ABC), limit = 30; for BA, minimum pulse frequency = 0, max. pulse frequency = 10.0, pulse rate = 0.95, noise = 0.8; for Biogeography-Based Optimization (BBO), mutation probability = 0.01, elite size = 2; for DE, weighting factor = 0.8, crossover rate = 0.9; for FA, light absorption coefficient = 0.001, mutation coefficient = 0.2, mutation step size = 0.05, exponent = 2; for Genetic Algorithm (GA), crossover probability = 0.95, mutation probability = 0.025; for Gradient-Based Optimizer (GBO), probability parameter = 0.5, min. beta = 0.2, max. beta = 1.2; for Grasshopper Optimization Algorithm (GOA), c min. coefficient = 0.00004, c max. coefficient = 1.0; for GWO, alpha, beta, delta, and omega coefficients; for Harris Hawks Optimization (HHO), energy initiation coefficient = [−1, 1); for ICA, empire count = 5, assimilation coefficient = 1.5, revolution probability = 0.05, revolution rate = 0.1; for IPA, donors = [1, 4], receivers = [2, 4]; for Moth-Flame optimization (MFO), shape parameter = −1 → −2 linear decrease, spiral frequency = 1, moth position = [−1, 1); for Manta Ray Foraging Optimization (MRFO), flip range = 2.0; for PSO, local coefficient = 2.05, global coefficient = 2.05, min. range = 0.4; for Sine Cosine Algorithm (SCA), search range = 2 → 0 linear decrease; for Salp Swarm Algorithm (SSA), safety threshold value = 0.8, producer number = 0.2, sparrow number = 0.1; for Teaching-Learning-based Optimization (TLBO), learning factor = 1 or 2; and for WOA, balance coefficient = 2 → 0 linear decrease, motion shape determining constant = 1.

## 5. Results and Discussion

In this study, the IPA-ANN model was compared with 18 metaheuristic algorithms on 9 datasets for classification problems. Evaluation tests were performed based on the PS and evaluation count parameters. The test results of the algorithms compared on the datasets are shown in detail in the tables. In the experiments conducted in this study, the algorithms were run in a single-fold validation. For each dataset, a single train–test partition (80% training, 20% test) was generated using a fixed random seed and kept identical across all 10 runs and for all compared algorithms, including the four IPA-ANN variants. Consequently, the reported mean, standard deviation, best, and worst test accuracy values reflect the stochastic variability inherent to each algorithm’s optimization process rather than variability introduced by different data partitions, thereby ensuring a controlled and directly comparable evaluation across algorithms. To ensure more consistent and fair results, each algorithm was run 10 times in a single-fold validation. The results demonstrating the mean, standard deviation, best and worst values, as well as F1-score metrics related to test accuracy, are presented in tables. Finally, confusion matrices and convergence graphs were presented in visual form. All tests were run on TÜBİTAK ULAKBİM High Performance and Grid Computing Center (TRUBA) resources (https://www.truba.gov.tr/, accessed on 1 April 2026).

In the experiments conducted in this study, four different donor–receiver parameters were evaluated for the IPA-ANN model: IPA_1_2 shows a donor value of 1 and a receiver value of 2; IPA_2_2 shows a donor value of 2 and a receiver value of 2; IPA_3_3 shows a donor value of 3 and a receiver value of 3; and IPA_4_4 shows a donor value of 4 and a receiver value of 4.

Table 3 shows that for DS1, the highest value among the IPAs is in the average-value section for IPA_3_3-ANN and IPA_4_4-ANN, while the best value is in IPA_3_3-ANN. In the average-value section, IPA_3_3-ANN and IPA_4_4-ANN achieved a test accuracy of 0.9443, outperforming all algorithms except GWO-ANN. In the best-value section, IPA_3_3-ANN achieved a value of 0.9571, the same as GWO-ANN. It achieved better test accuracy than all other algorithms. In the standard deviation section, IPA_4_4-ANN achieved the same value as GWO-ANN. It showed more stable performance than all other algorithms. In the F1-score section, IPA_3_3-ANN was seen to be better than all algorithms. For DS2, IPA_3_3-ANN achieved the best values among the IPAs in both the average-value and best-value sections. In the average-value section, IPA_3_3-ANN achieved a value of 0.8009, which was better than 12 algorithms. It has the same value as PSO-ANN. In the best-value section, IPA_3_3-ANN achieved better results than 13 algorithms with a value of 0.8141. It also has the same value as three algorithms. In the standard deviation section, IPA_4_4-ANN showed more stable performance than the other algorithms, except for BA-ANN. In the F1-score section, IPA_3_3-ANN was seen to be better than all others, except for GWO-ANN. For DS3, the highest values in the average and best-value sections among the IPAs were observed in IPA_1_2-ANN and IPA_3_3-ANN. In the average-value section, IPA_1_2-ANN and IPA_3_3-ANN achieved a test accuracy of 0.5899, outperforming all algorithms except MRFO-ANN. In the best-value section, IPA_1_2-ANN and IPA_3_3-ANN achieved a value of 0.6014, the same as three algorithms. They achieved better test accuracy than the remaining algorithms. In the standard deviation section, IPA_1_2-ANN showed more stable performance than nine algorithms. In the F1-score section, IPA_1_2-ANN achieved better results than all algorithms.

Table 4 shows that for DS4, the highest test accuracy among the IPAs was observed in the IPA_1_2-ANN for both the average-value and best-value sections. In the average-value section, IPA_1_2-ANN achieved a value of 0.8885, outperforming all other algorithms. In the best-value section, IPA_1_2-ANN achieved the same value as the GWO-ANN algorithm, with a value of 0.9038. It was seen to achieve better results than all other algorithms. In the standard deviation section, IPA_2_2-ANN achieved the same value as three algorithms. It showed more stable performance than the remaining algorithms. In the F1-score section, IPA_1_2-ANN achieved better results than all others except GWO-ANN. Among the IPAs for DS5, IPA_4_4-ANN was found to be the best in the average-value section, while IPA_2_2-ANN and IPA_4_4-ANN were the best in the best-value section. In the average-value section, IPA_4_4-ANN achieved better results than 11 algorithms with a value of 0.6459. It also achieved the same value as the GBO-ANN and PSO-ANN algorithms. In the best-value section, IPA_2_2-ANN and IPA_4_4-ANN achieved the same value as four algorithms, with a value of 0.6721. It was also seen to be better than the other algorithms except GBO-ANN. In the standard deviation section, IPA_2_2-ANN has the same value as two algorithms. It showed more stable performance than the remaining six algorithms. In the F1-score section, IPA_2_2-ANN achieved the same value as DE-ANN and SCA-ANN. It obtained better results than the other algorithms except for GBO-ANN and ABC-ANN. Among the IPAs for DS6, IPA_3_3-ANN achieved the best results in the average-value and best-value sections. In the average-value section, IPA_3_3-ANN was seen to be better than all algorithms with a value of 0.8258. In the best-value section, IPA_3_3-ANN also achieved the same value as the BBO-ANN algorithm, with a value of 0.8710. It achieved better test accuracy than the remaining algorithms. In the standard deviation section, IPA_2_2-ANN showed more stable performance than 15 algorithms. In the F1-score section, IPA_3_3-ANN achieved better results than all other algorithms.

Table 5 shows that for DS7, the best values among the IPAs are found in the average-value section of IPA_3_3-ANN, and in the best-value section of IPA_2_2-ANN and IPA_3_3-ANN. In the average-value section, IPA_3_3-ANN is seen to be better than all algorithms with a value of 0.9667. In the best-value section, IPA_2_2-ANN and IPA_3_3-ANN have the same value as the GA-ANN algorithm, with a value of 1.0000. It obtained better results than all the remaining algorithms. In the standard deviation section, IPA_4_4-ANN showed more stable performance than nine algorithms. In the F1-score section, the same results as in the best-value section were observed. For DS8, among the IPAs, the best values were seen in the average-value section for IPA_3_3-ANN and in the best-value section for IPA_1_2-ANN. In the average-value section, IPA_3_3-ANN was seen to have a better test accuracy than seven algorithms, with a value of 0.6594. It has the same value as the PSO-ANN algorithm. In the best-value section, IPA_1_2-ANN was seen to be better than all algorithms except GWO-ANN and HHO-ANN, with a value of 0.7135. In the standard deviation section, IPA_3_3-ANN showed more stable performance than all other algorithms. In the F1-score section, IPA_1_2-ANN achieved better results than all other algorithms except GWO-ANN and HHO-ANN. It also achieved the same value as the GA-ANN algorithm. For DS9, among the IPAs, the best values in the average-value section were found in IPA_4_4-ANN, while the best values in the best-value section were found in IPA_2_2-ANN and IPA_3_3-ANN. In the average-value section, IPA_4_4-ANN achieved the same value as the PSO-ANN algorithm, with a value of 0.9172. It was observed to be better than 10 of the remaining algorithms. In the best-value section, IPA_2_2-ANN and IPA_3_3-ANN achieved the same value as four algorithms with a value of 0.9425. It achieved better test accuracy than the remaining algorithms except MRFO-ANN. In the standard deviation section, IPA_4_4-ANN achieved the same value as PSO-ANN. It showed more stable performance than the remaining 12 algorithms. In the F1-score section, IPA_2_2-ANN and IPA_3_3-ANN achieved the same value as the BBO-ANN and DE-ANN algorithms. Better values were obtained than all algorithms except MRFO-ANN.

When all tables are examined, the ranking based on the best test accuracy values shows IPA-ANN in first place with an average of 1.66; GWO-ANN in second place with an average of 2; DE-ANN in third place with an average of 2.33; PSO-ANN in fourth place with an average of 2.66; and TLBO-ANN in fifth place with an average of 2.77.

**Figure 6:**
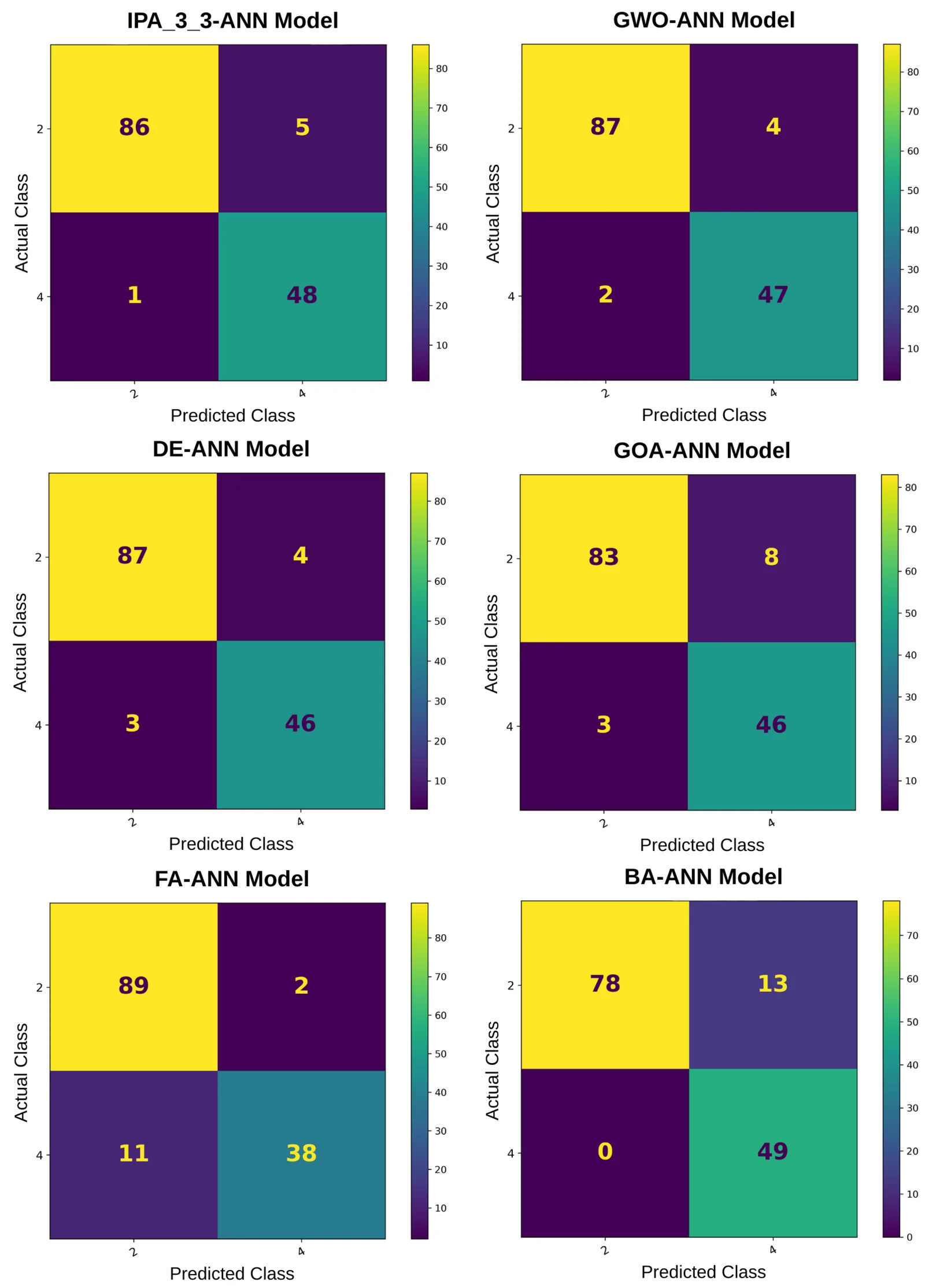
Confusion matrices of the 3 best and worst algorithms in DS1.

Figure 6 shows the confusion matrices for the three best and worst algorithms in DS1. The best test-accuracy values for the models were obtained using the confusion matrix. The class labels in the matrix are represented as 2 for benign and 4 for malignant. The matrix shows the numbers TN in the upper left, FP in the upper right, FN in the lower left, and TP in the lower right. IPA_3_3-ANN and GWO-ANN made 6 misclassifications; DE-ANN made 7; GOA-ANN made 11; and FA-ANN and BA-ANN made 13 misclassifications.

**Figure 7:**
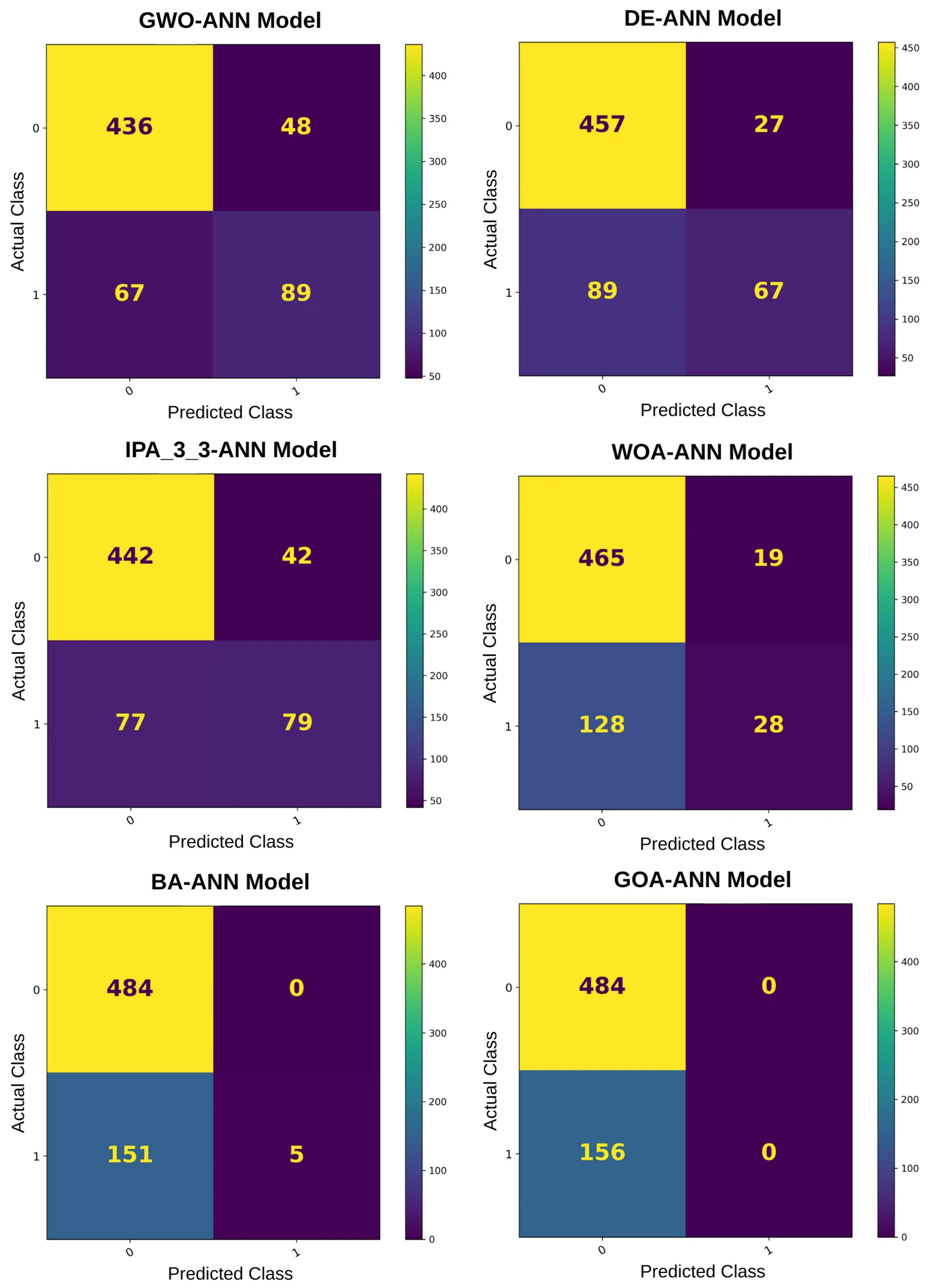
Confusion matrices of the 3 best and worst algorithms in DS2.

Figure 7 shows the confusion matrices for the three best and worst algorithms in DS2. The best test-accuracy values for the models were obtained using the confusion matrix. In the matrix, class labels 0 indicate that the whites won; 1 indicates that the whites lost. The matrix shows the numbers TN in the upper left, FP in the upper right, FN in the lower left, and TP in the lower right. GWO-ANN made 115 misclassifications; DE-ANN made 116; IPA_3_3-ANN made 119; WOA-ANN made 147; BA-ANN made 151; and GOA-ANN made 156 misclassifications.

**Figure 8:**
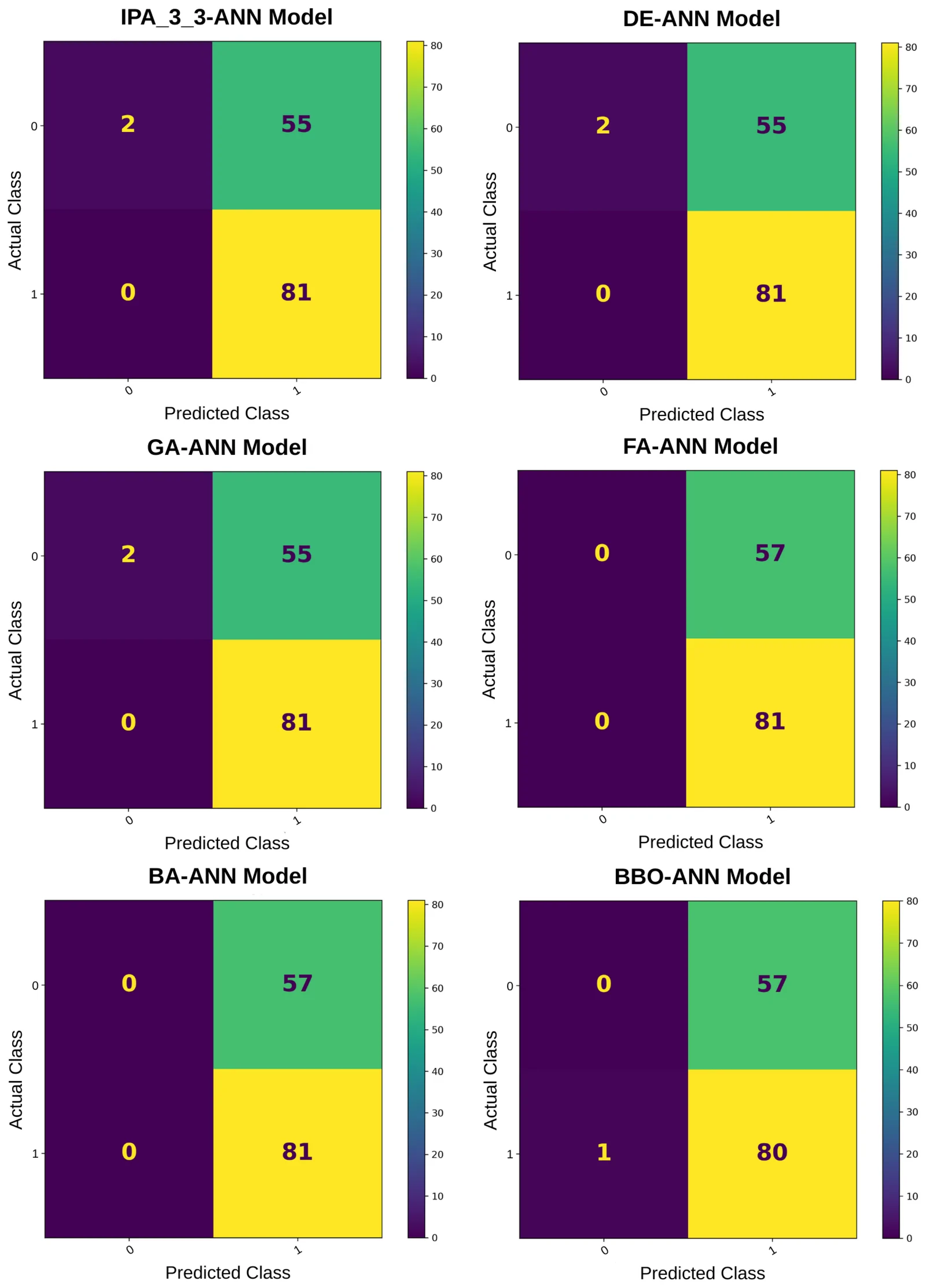
Confusion matrices of the 3 best and worst algorithms in DS3.

Figure 8 shows the confusion matrices for the three best and worst algorithms in DS3. The best test-accuracy values for the models were obtained using the confusion matrix. In the matrix, class labels 0 indicate that the credit was rejected, and 1 indicates that the credit was approved. The matrix shows the numbers TN in the upper left, FP in the upper right, FN in the lower left, and TP in the lower right. IPA_3_3-ANN, DE-ANN, and GA-ANN made 55 misclassifications; FA-ANN and BA-ANN made 57; and BBO-ANN made 58.

**Figure 9:**
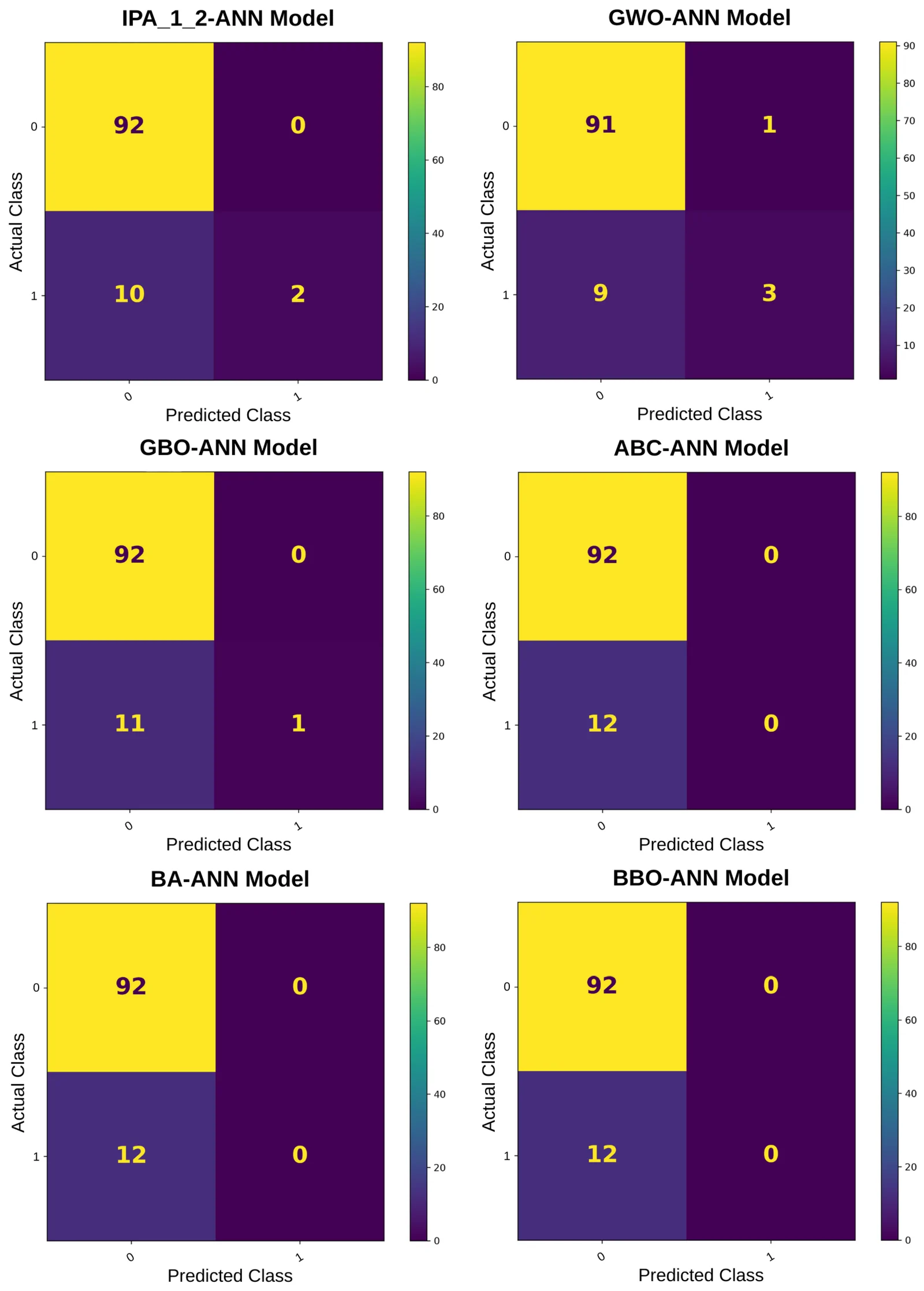
Confusion matrices of the 3 best and worst algorithms in DS4.

Figure 9 shows the confusion matrices for the three best and worst algorithms in DS4. The best test-accuracy values for the models were obtained using the confusion matrix. In the matrix, class labels 0 indicate patients without diabetes risk, and 1 indicates patients with diabetes risk. The matrix shows the numbers of TN in the upper left, FP in the upper right, FN in the lower left, and TP in the lower right. IPA_1_2-ANN and GWO-ANN made 10 misclassifications; GBO-ANN made 11; and ABC-ANN, BA-ANN, and BBO-ANN made 12 misclassifications.

**Figure 10:**
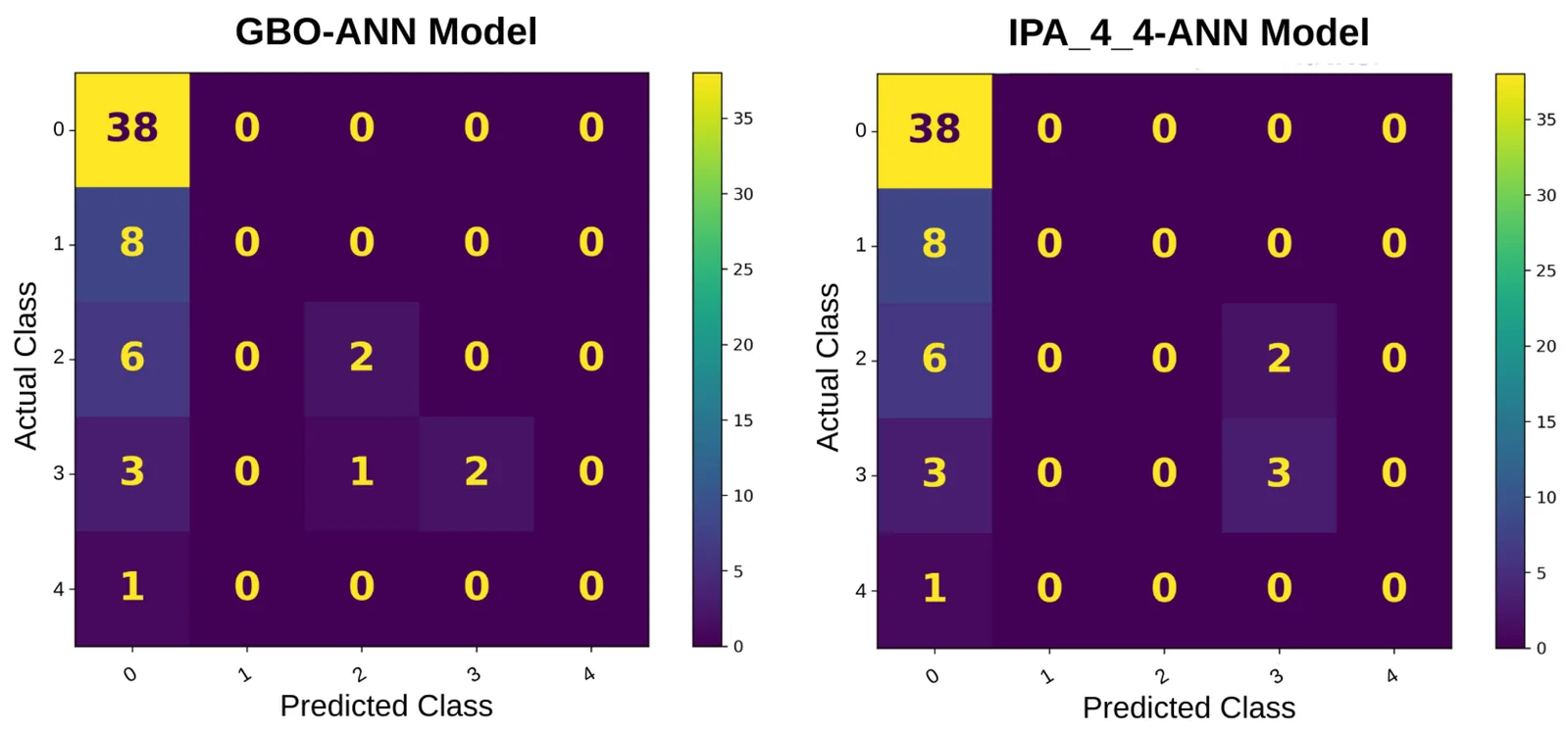

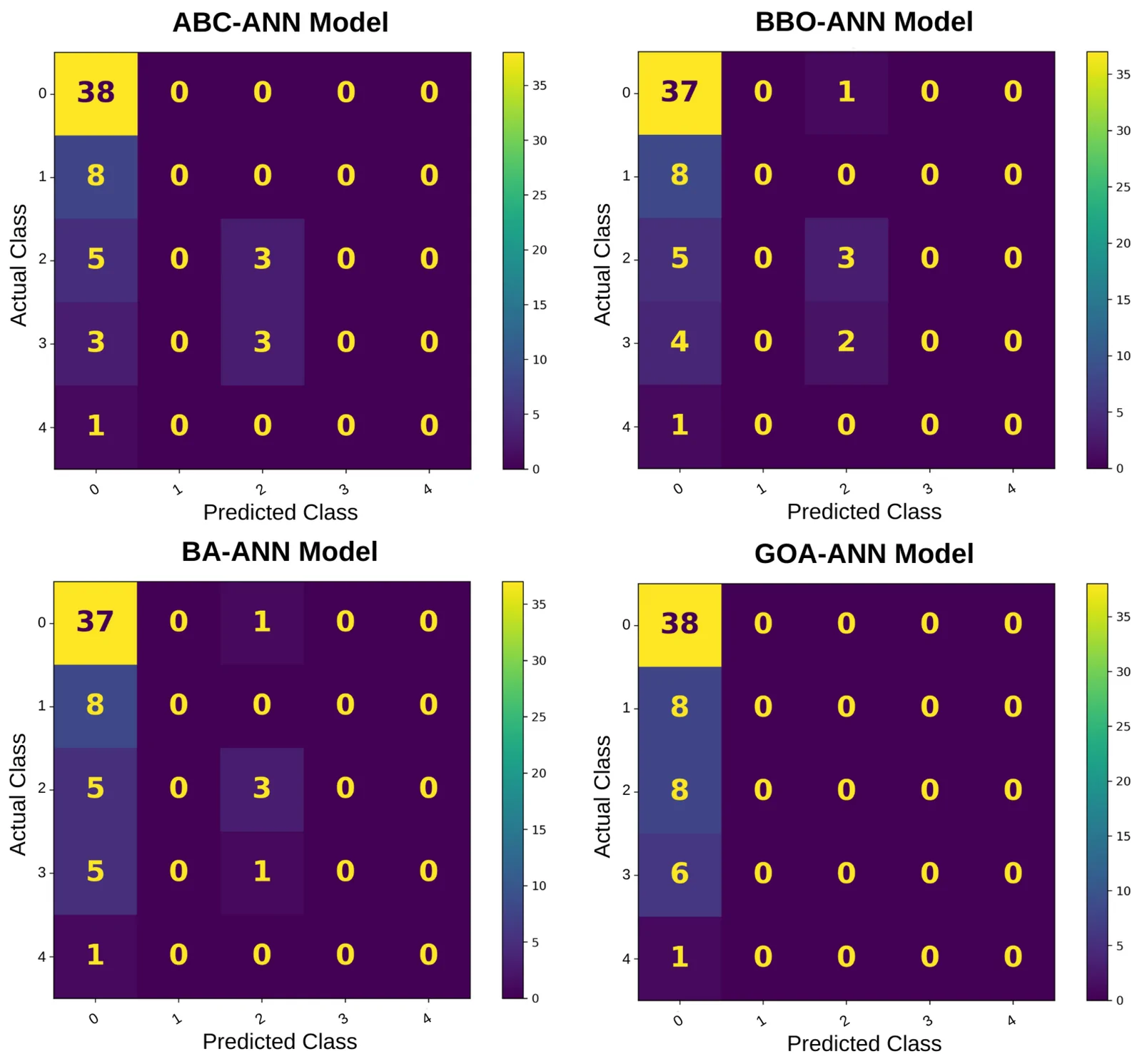
Confusion matrices of the 3 best and worst algorithms in DS5.

Figure 10 shows the confusion matrices for the three best and worst algorithms in DS5. The best test-accuracy values for the models were obtained using the confusion matrix. In the matrix, class labels 0 indicate no disease, 1 mild disease, 2 moderate disease, 3 serious disease, and 4 very serious disease. GBO-ANN made 19 misclassifications; IPA_4_4-ANN and ABC-ANN made 20; BBO-ANN and BA-ANN made 21; and GOA-ANN made 23 misclassifications.

**Figure 11:**
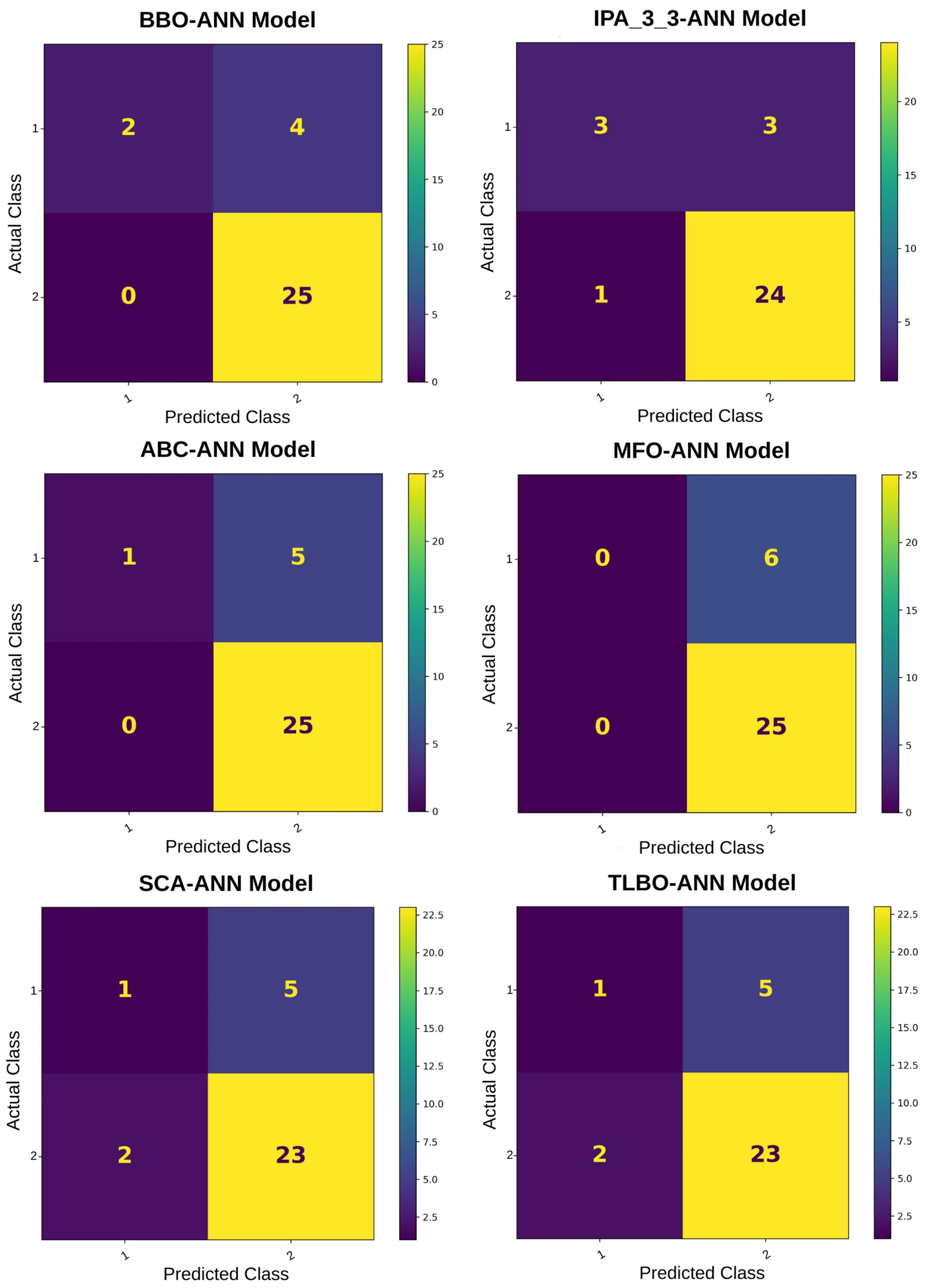
Confusion matrices of the 3 best and worst algorithms in DS6.

Figure 11 shows the confusion matrices for the three best and worst algorithms in DS6. The best test-accuracy values for the models were obtained using the confusion matrix. In the matrix, class labels 1 and 2 represent living and deceased patients, respectively. The matrix shows the numbers of TN in the upper left, FP in the upper right, FN in the lower left, and TP in the lower right. BBO-ANN and IPA_3_3-ANN made four misclassifications; ABC-ANN made five; MFO-ANN made six; and SCA-ANN and TLBO-ANN made seven misclassifications.

**Figure 12:**
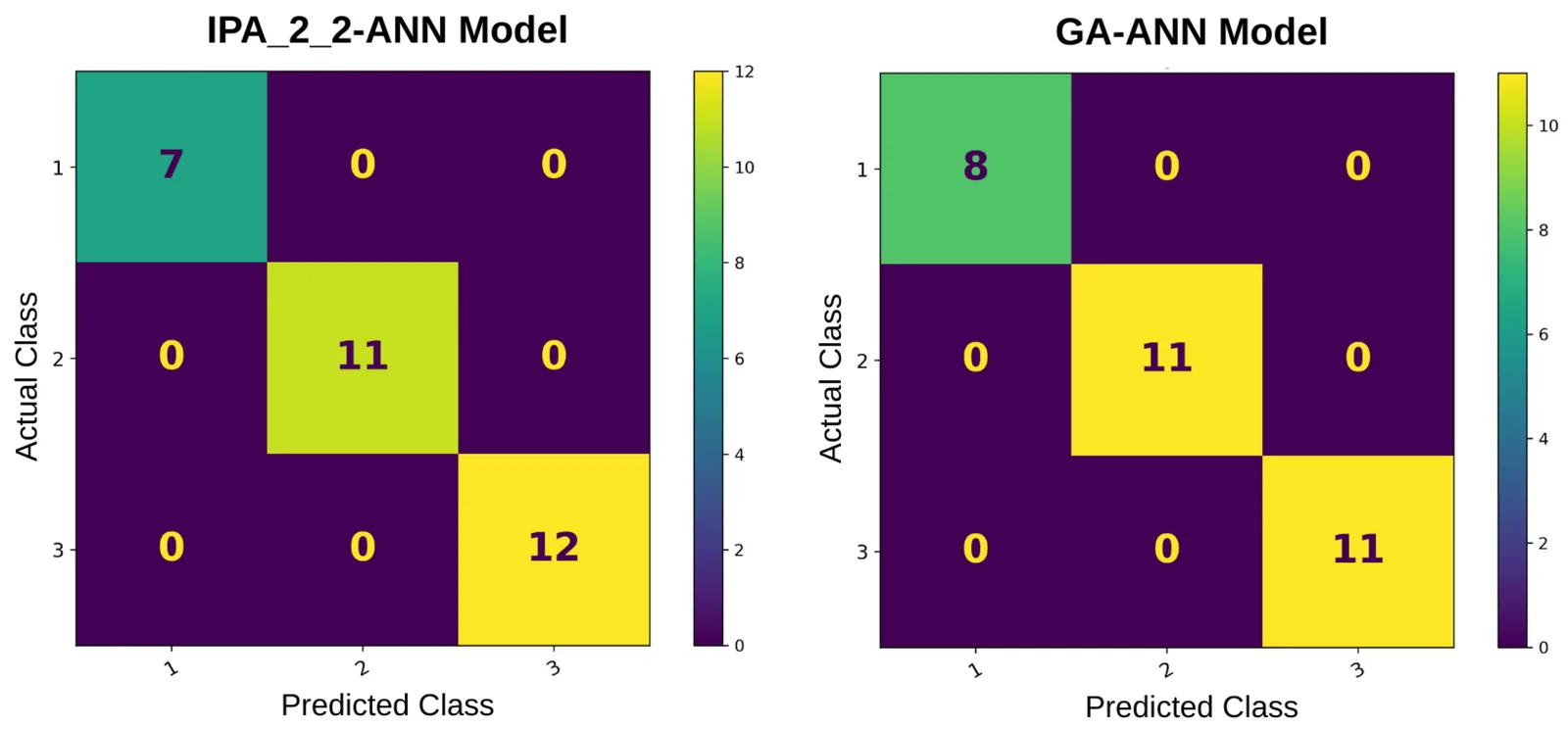

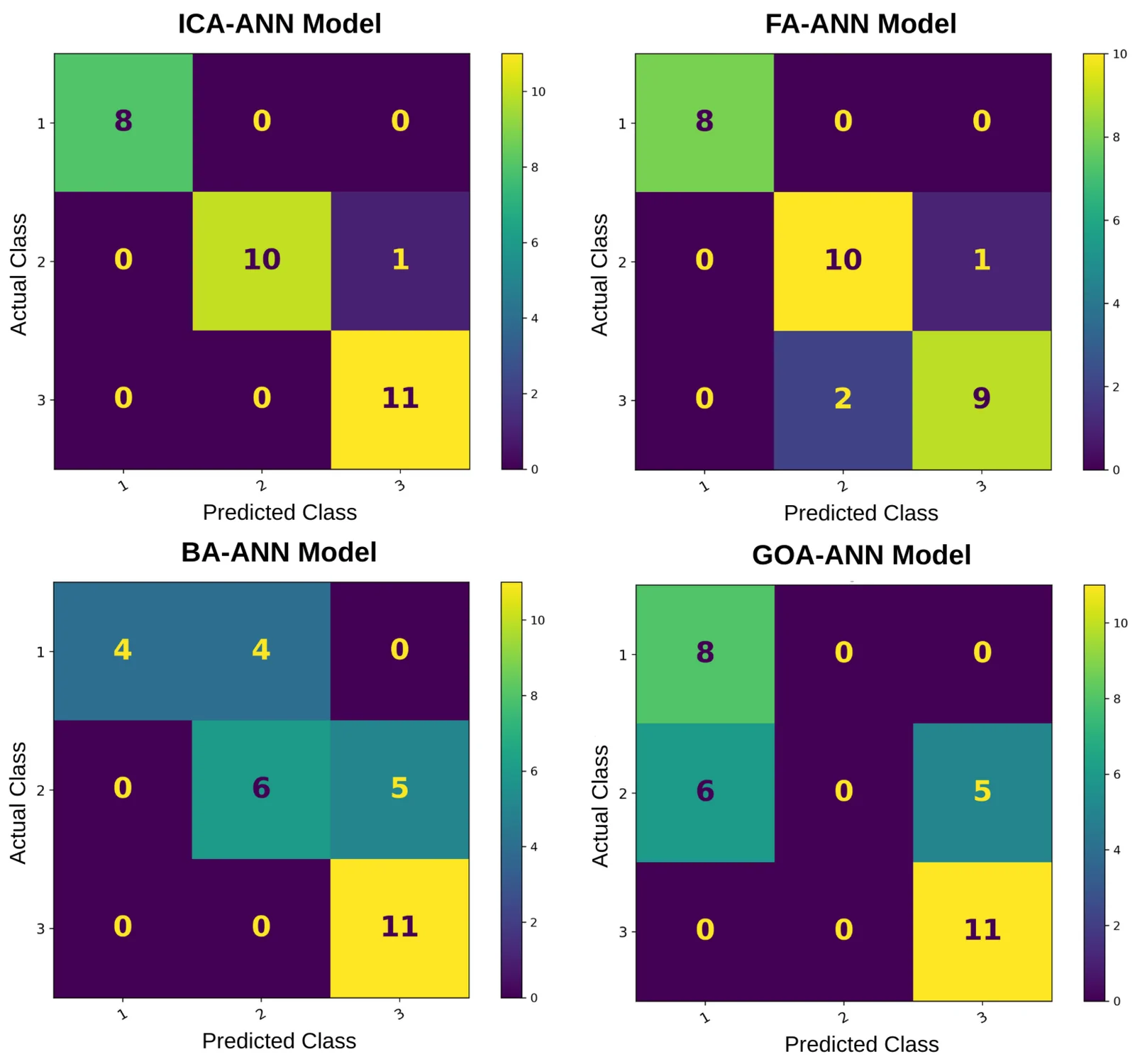
Confusion matrices of the 3 best and worst algorithms in DS7.

Figure 12 shows the confusion matrices for the three best and worst algorithms in DS7. The best test-accuracy values for the models were obtained using the confusion matrix. The class labels in the matrix are 1, iris-setosa; 2, iris-versicolor; 3, iris-virginica. IPA_2_2-ANN and GA-ANN made 0 misclassifications; ICA-ANN made 1; FA-ANN made 3; BA-ANN made 9; and GOA-ANN made 11 misclassifications.

**Figure 13:**
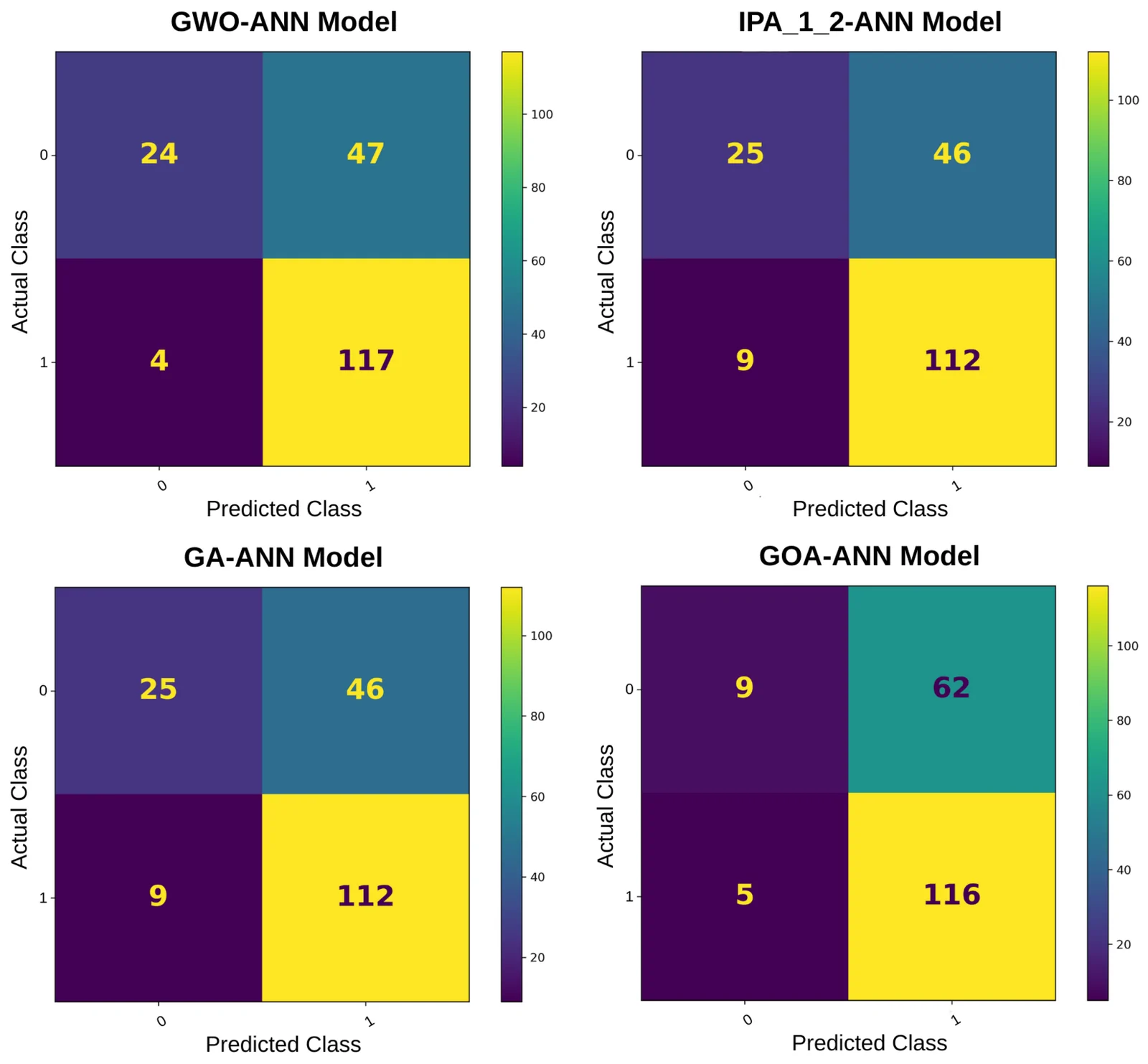

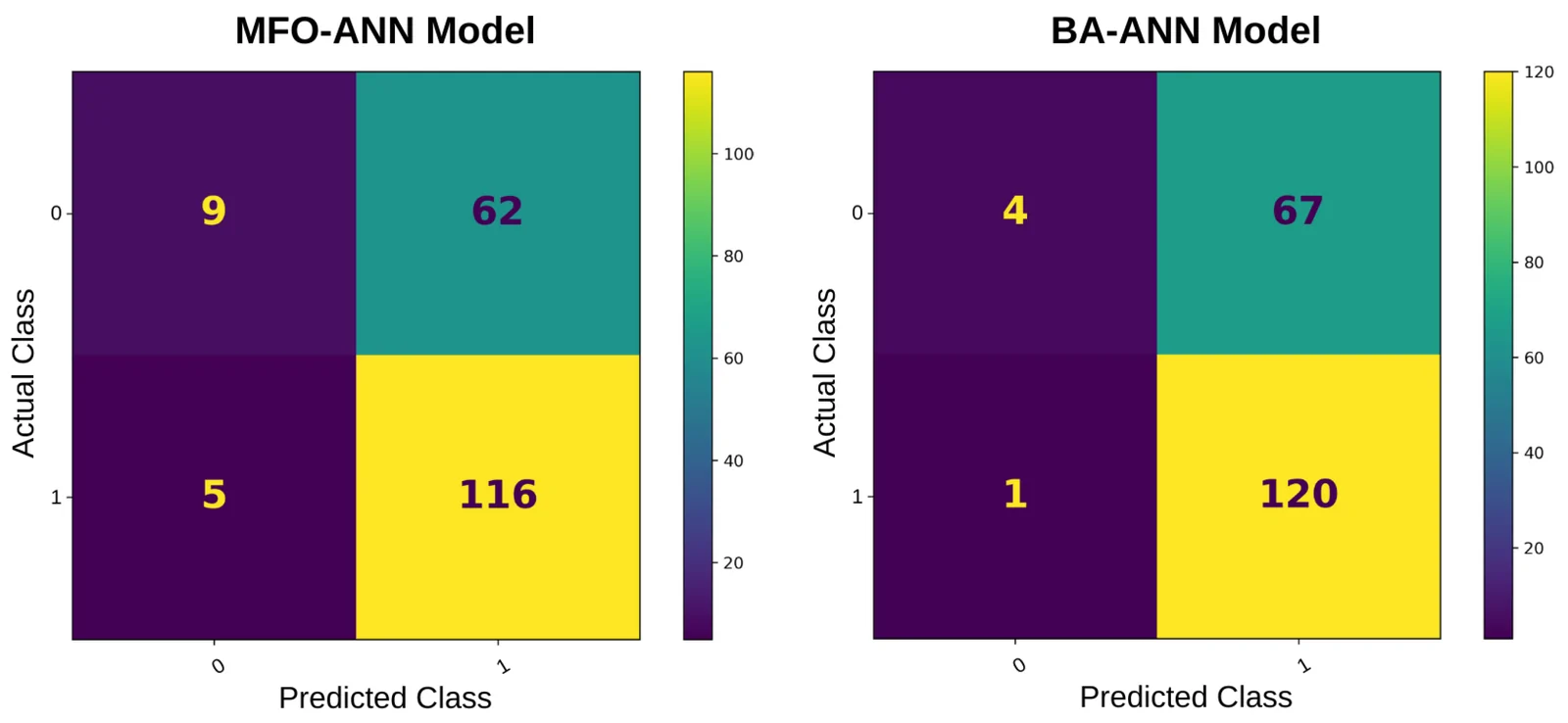
Confusion matrices of the 3 best and worst algorithms in DS8.

Figure 13 shows the confusion matrices for the three best and worst algorithms in DS8. The best test-accuracy values for the models were obtained using the confusion matrix. In the matrix, the class labels are 0, X did not win; 1, X won. The matrix shows the numbers TN in the upper left, FP in the upper right, FN in the lower left, and TP in the lower right. GWO-ANN made 51 misclassifications; IPA_1_2-ANN and GA-ANN made 55; GOA-ANN and MFO-ANN made 67; and BA-ANN made 68 misclassifications.

**Figure 14:**
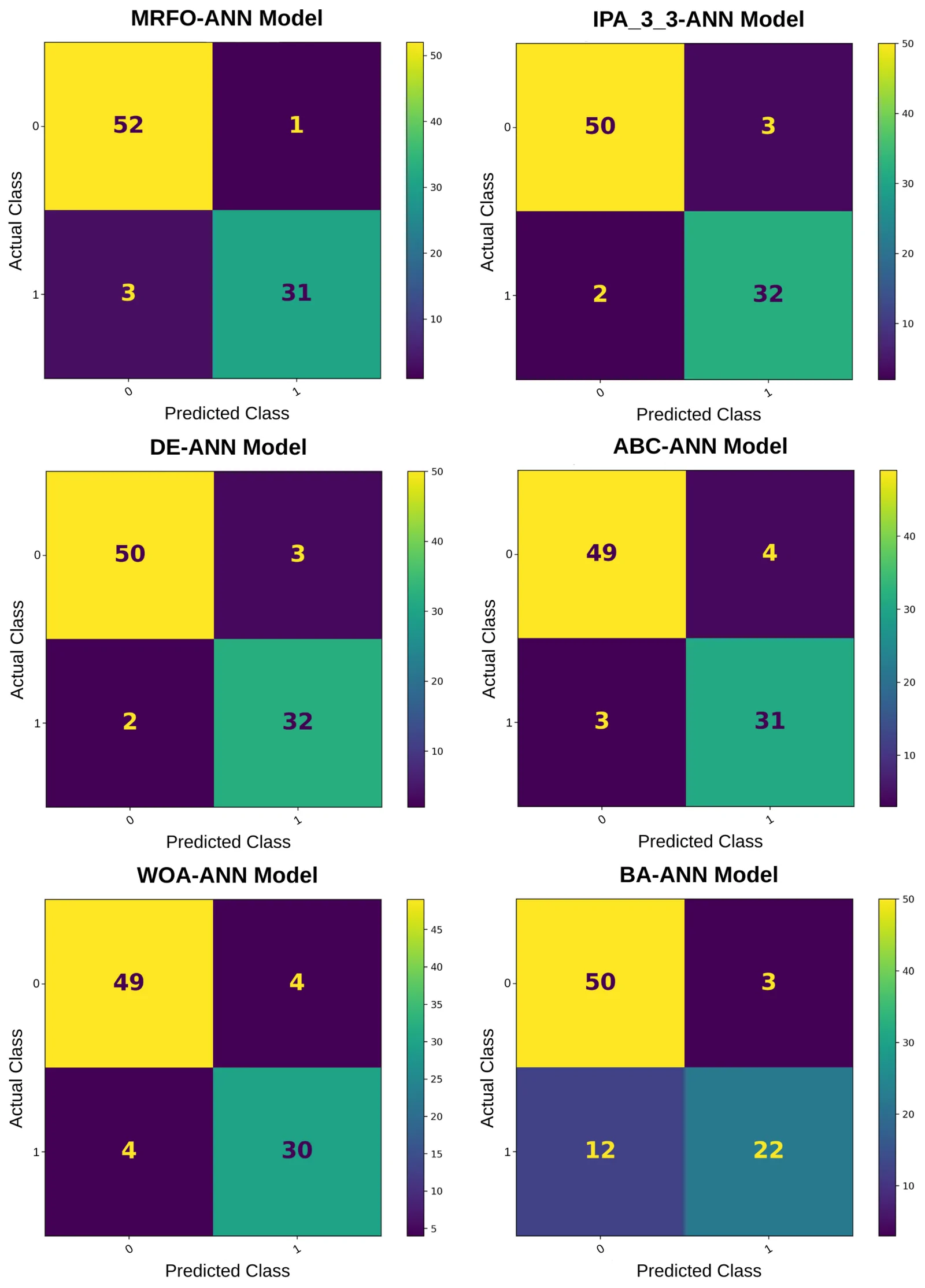
Confusion matrices of the 3 best and worst algorithms in DS9.

Figure 14 shows the confusion matrices for the three best and worst algorithms in DS9. The best test-accuracy values for the models were obtained using the confusion matrix. In the matrix, the class labels are 0 for Republican and 1 for Democrat. The matrix shows the numbers TN in the upper left, FP in the upper right, FN in the lower left, and TP in the lower right. MRFO-ANN made 4 misclassifications; IPA_3_3-ANN and DE-ANN made 5; ABC-ANN made 7; WOA-ANN made 8; and BA-ANN made 15 misclassifications.

**Figure 15:**
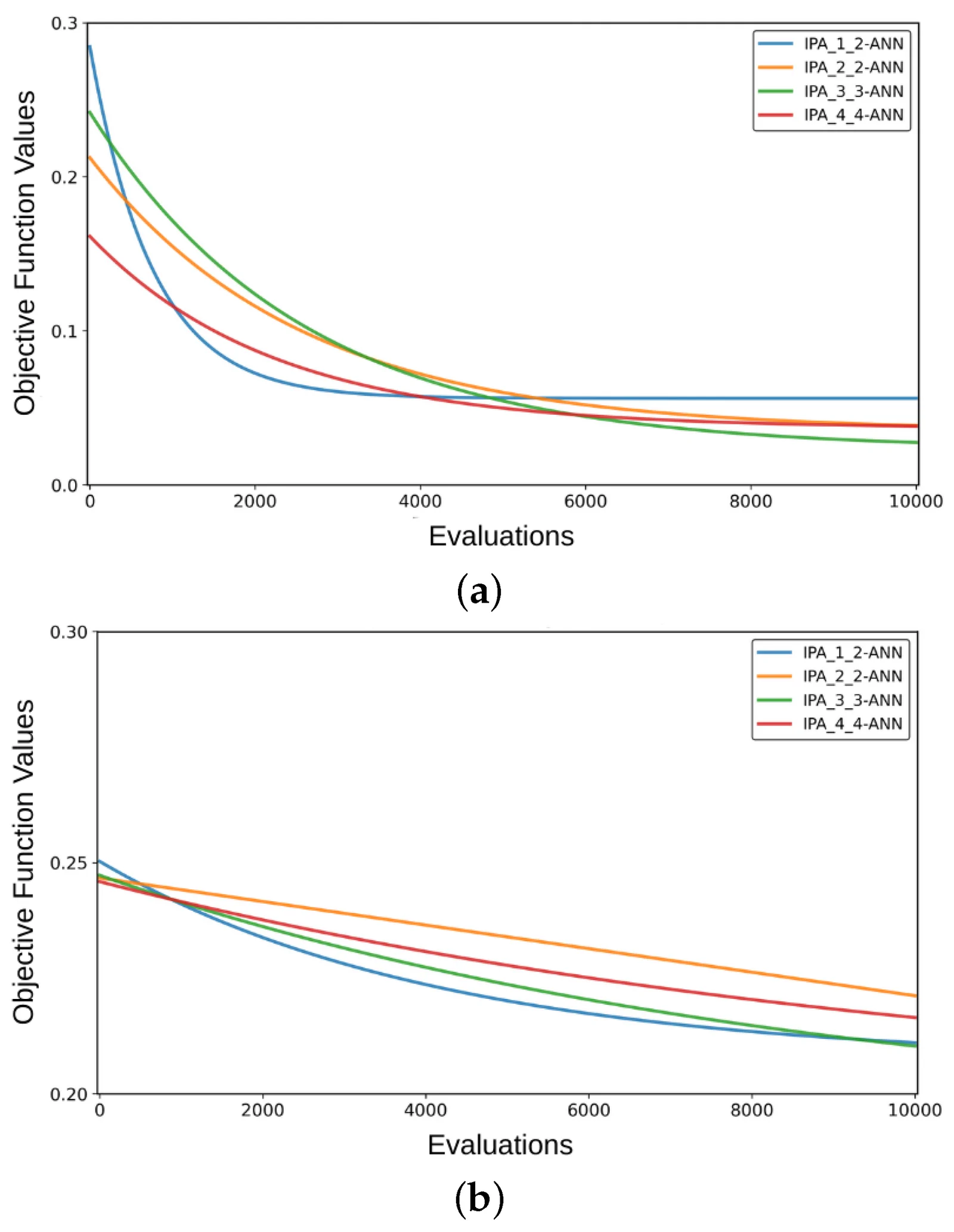

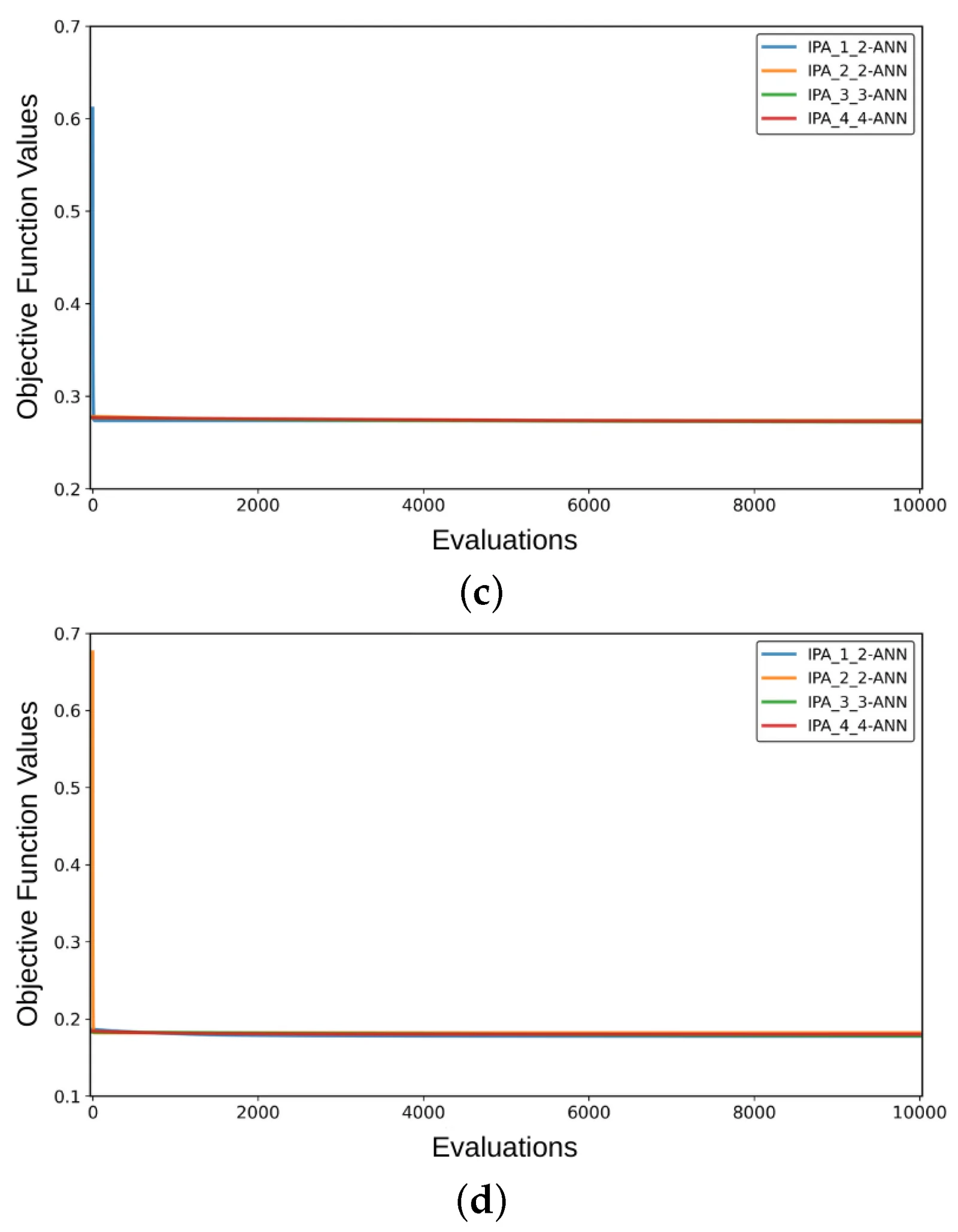
IPA-ANN convergence graphs (DS1–DS4). (**a**) DS1, (**b**) DS2, (**c**) DS3, (**d**) DS4.

**Figure 16:**
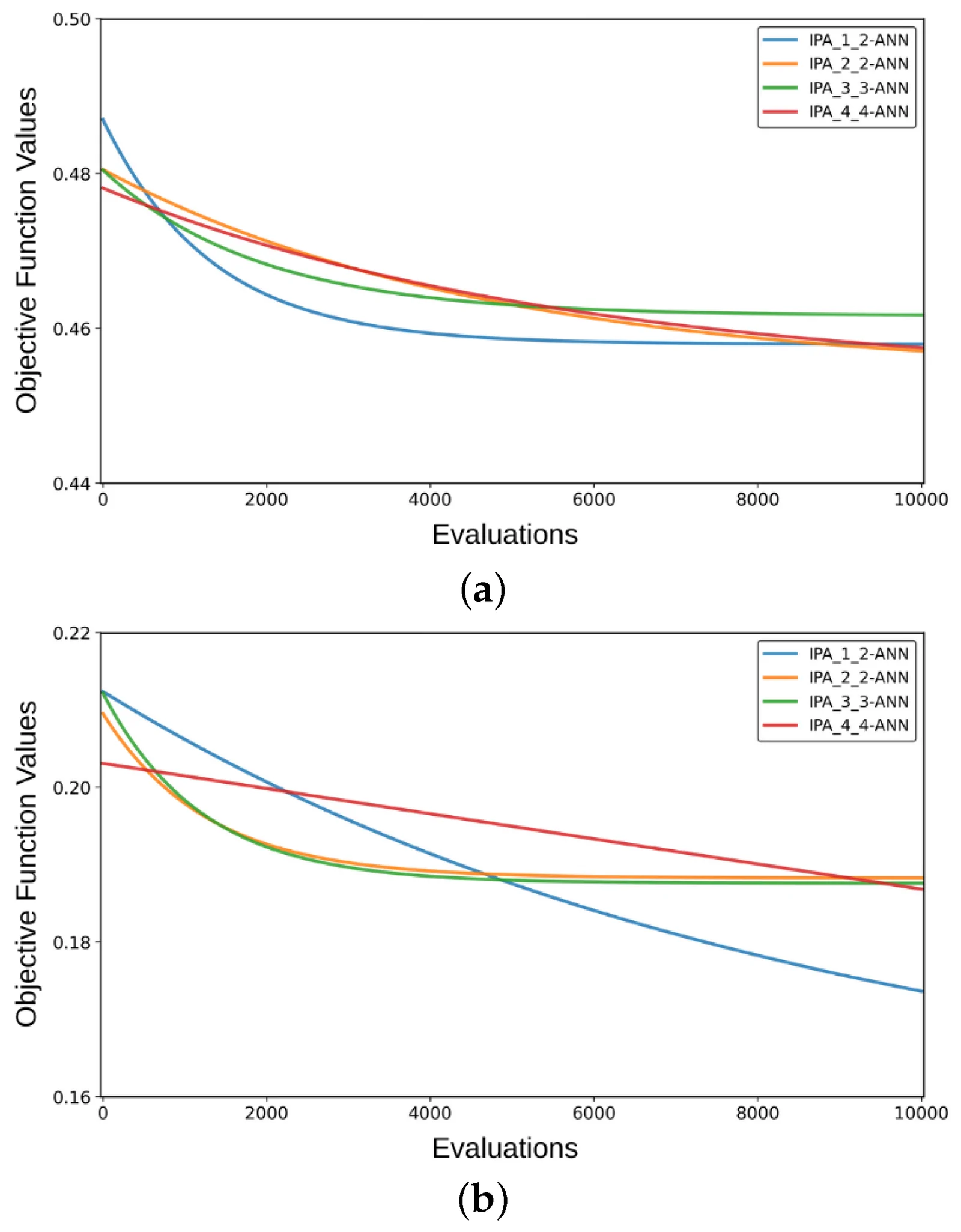

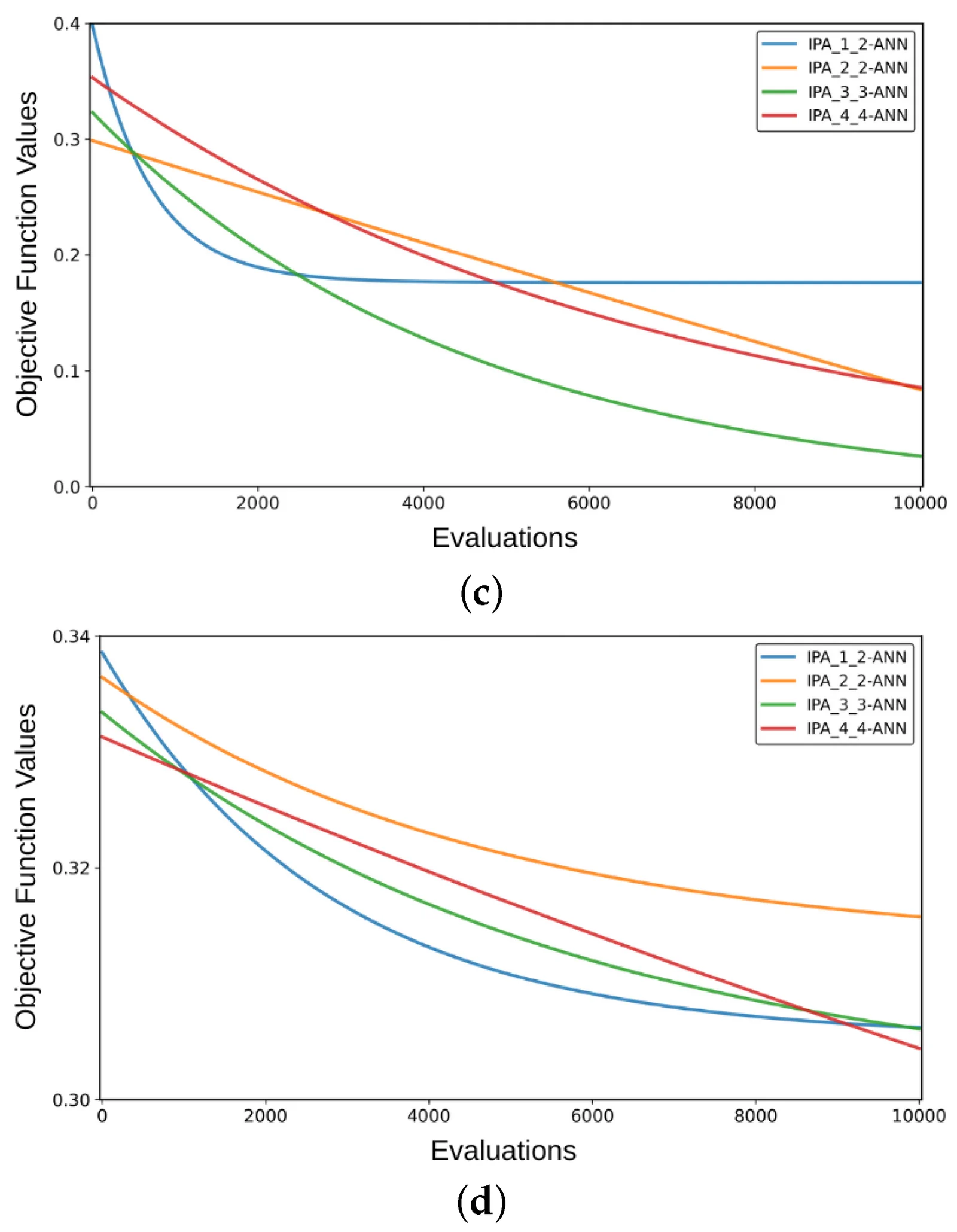
IPA-ANN convergence graphs (DS5–DS8). (**a**) DS5, (**b**) DS6, (**c**) DS7, (**d**) DS8.

**Figure 17:**
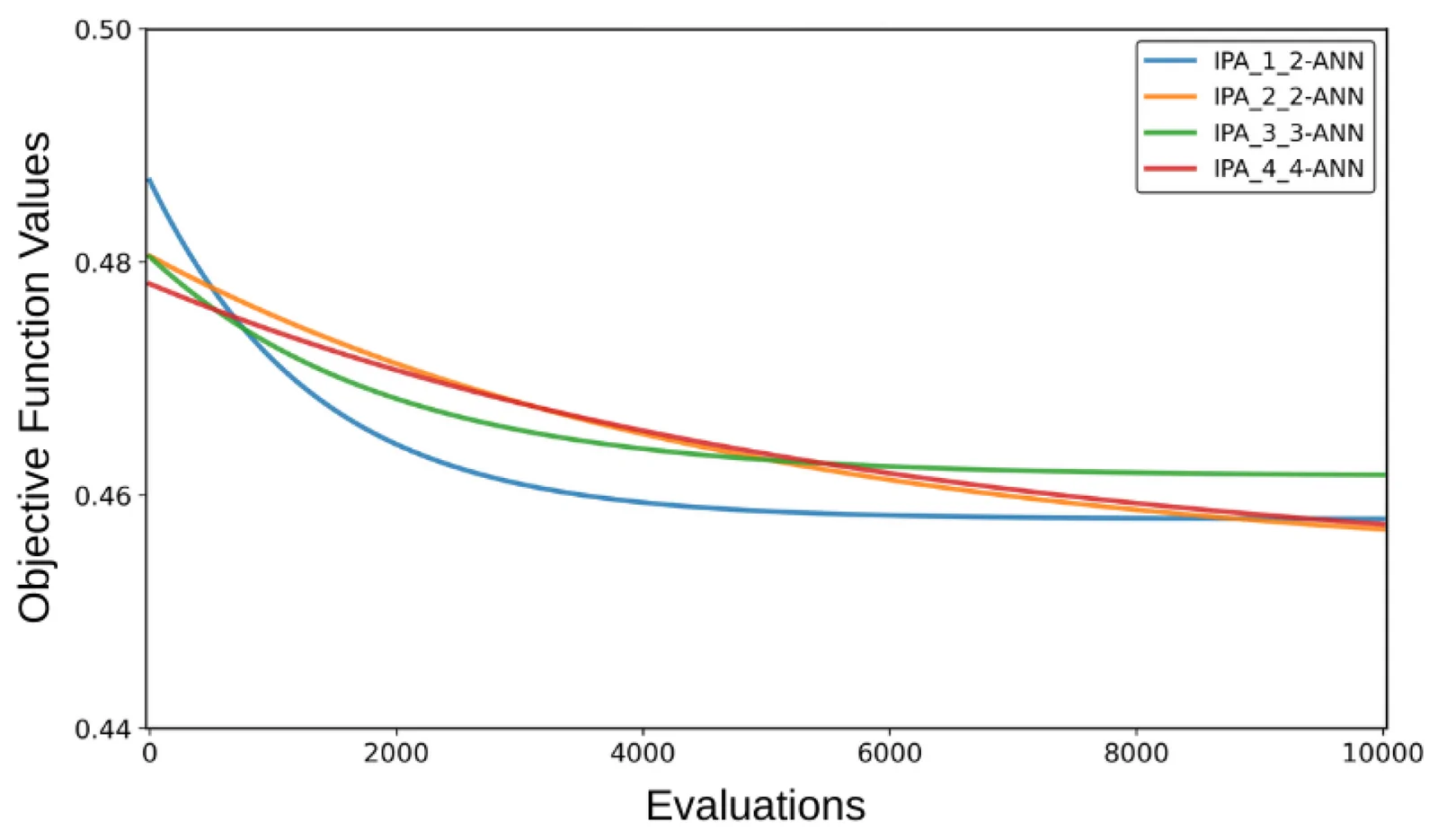
IPA-ANN convergence graph (DS9).

The convergence plots for the parameters donor = 1 and receiver = 2, donor = 2 and receiver = 2, donor = 3 and receiver = 3, and donor = 4 and receiver = 4 in IPA-ANN for DS1-DS9 are examined in Figure 15, Figure 16 and Figure 17. Upon examining the graphs, for DS1, IPA_1_2-ANN converged faster at 2000 evaluations; IPA_4_4-ANN at 4000 evaluations; and IPA_3_3-ANN at 6000, 8000, and 10,000 evaluations. For DS2, IPA_1_2-ANN converged faster in 2000, 4000, 6000, and 8000 evaluations; IPA_3_3-ANN converged faster in 10,000 evaluations. For DS3, IPA_3_3-ANN converged faster at 2000, 4000, 6000, and 8000 evaluations; IPA_4_4-ANN converged faster at 10,000 evaluations. For DS4, IPA_1_2-ANN converged faster at 2000, 4000, 6000, and 8000 evaluations; IPA_3_3-ANN converged faster at 10,000 evaluations. For DS5, IPA_1_2-ANN converged faster at 2000, 4000, 6000, and 8000 evaluations; IPA_2_2-ANN converged faster at 10,000 evaluations. For DS6, IPA_3_3-ANN converged faster in 2000 and 4000 evaluations; IPA_1_2-ANN converged faster in 6000, 8000, and 10,000 evaluations. For DS7, IPA_1_2-ANN converged faster at 2000 evaluations; IPA_3_3-ANN converged faster in the remaining evaluations. For DS8, IPA_1_2-ANN converged faster in 2000, 4000, 6000, and 8000 evaluations; IPA_4_4-ANN converged faster in 10,000 evaluations. For DS9, IPA_1_2-ANN converged faster in 2000 and 4000 evaluations; IPA_2_2-ANN in 6000 evaluations; IPA_3_3-ANN in 8000 evaluations; and IPA_4_4-ANN in 10,000 evaluations.

Based on the results, IPA_1_2-ANN achieved faster convergence in 2000, 4000, and 6000 evaluations, while IPA_3_3-ANN and IPA_4_4-ANN exhibited better convergence behavior in high evaluations for some datasets. Specifically, in the DS2, DS4, DS5, and DS9 datasets, IPA_1_2-ANN achieved superior convergence performance in early evaluations. In other datasets, convergence performance varies depending on the specific dataset.

These results reveal that the donor–receiver parameters of the IPA have a significant effect on convergence performance and that the correct parameter selection varies depending on the dataset. Overall, while IPA_1_2-ANN provides fast convergence and consistent performance in the early stages, IPA_3_3-ANN and IPA_4_4-ANN achieve lower objective function values in some datasets.

**Figure 18:**
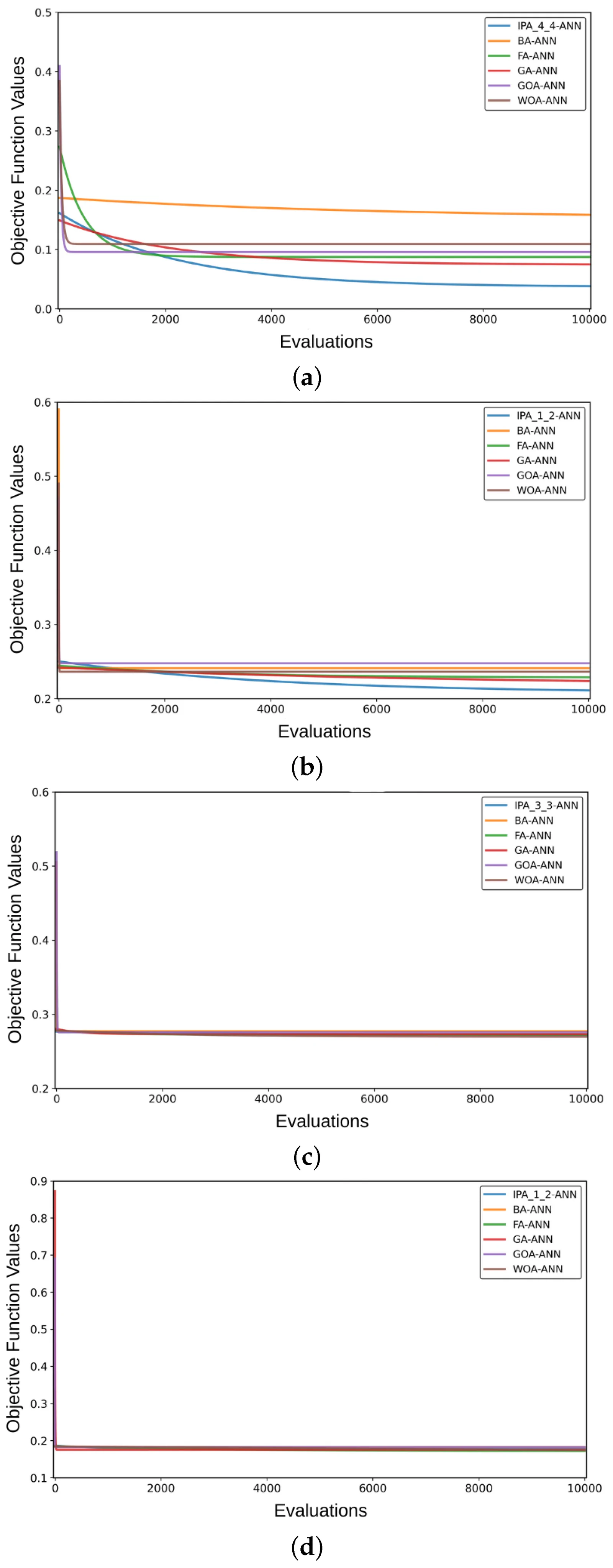
Convergence graphs of sample algorithms compared with IPA-ANN (DS1–DS4). (**a**) DS1, (**b**) DS2, (**c**) DS3, (**d**) DS4.

**Figure 19:**
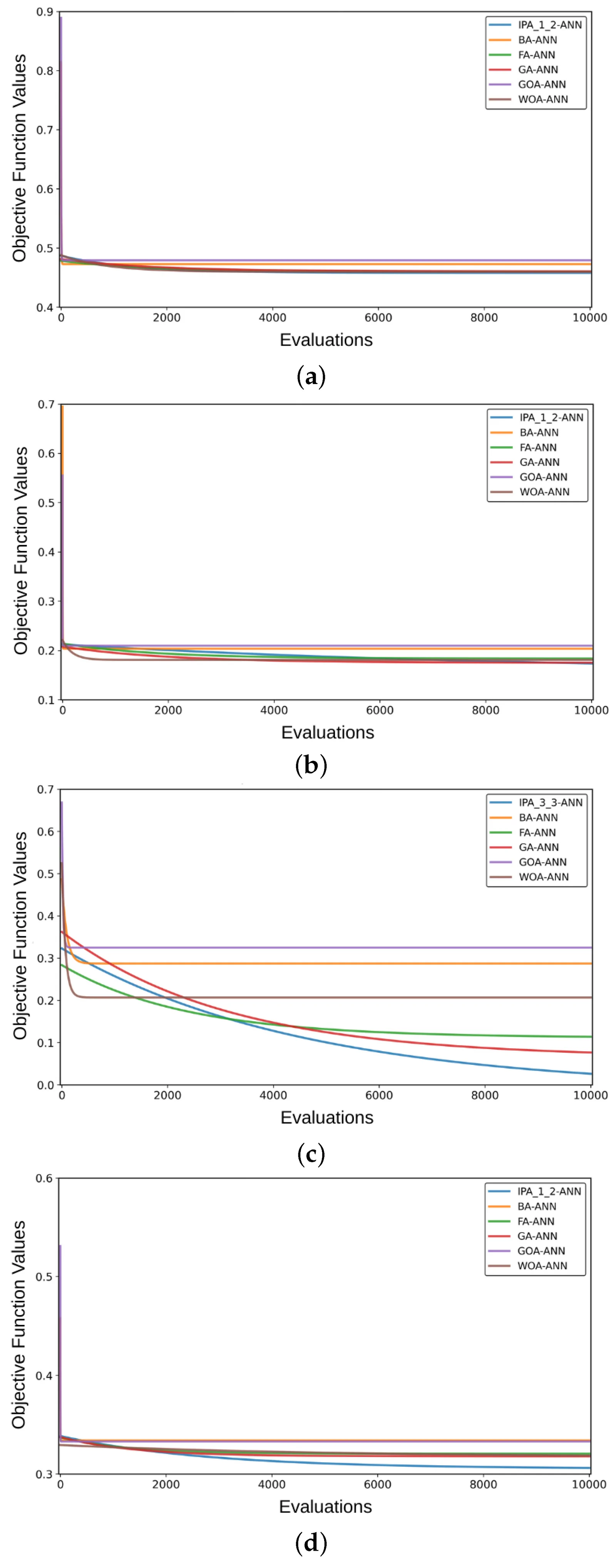
Convergence graphs of sample algorithms compared with IPA-ANN (DS5–DS8). (**a**) DS5, (**b**) DS6, (**c**) DS7, (**d**) DS8.

**Figure 20:**
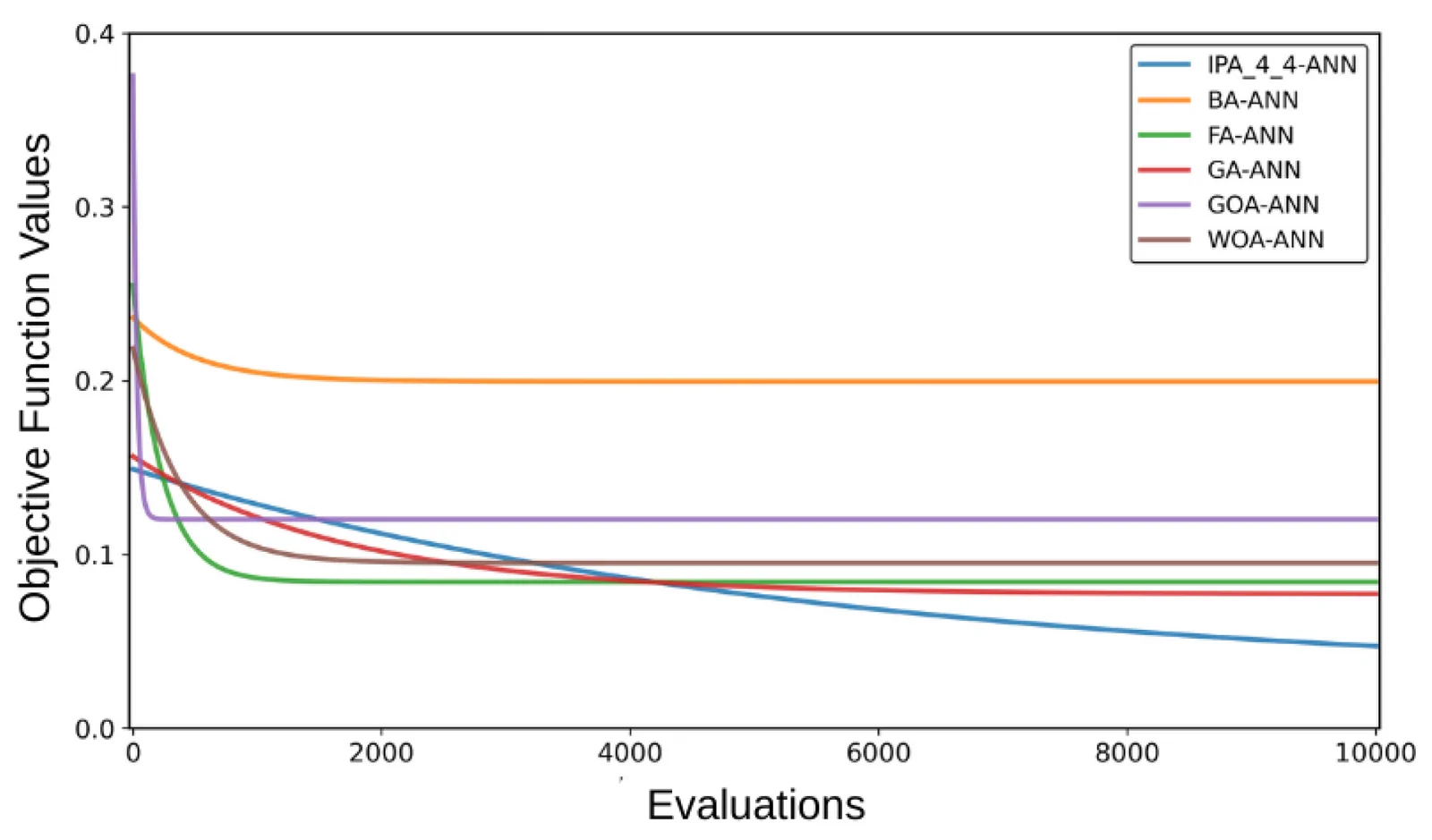
Convergence graphs of sample algorithms compared with IPA-ANN (DS9).

The convergence graphs of five randomly selected algorithms without using IPA-ANN in DS1-DS9 are examined in Figure 18, Figure 19 and Figure 20. Upon detailed examination of the figures, IPA-ANN provided faster convergence than the other algorithms in all evaluations for DS1. For DS2, IPA-ANN also converged faster than the compared algorithms in all evaluations. For DS3, the WOA-ANN algorithm provided the best convergence in all evaluations. IPA-ANN converged faster than FA-ANN in 2000, 4000, and 6000 evaluations, and faster than GA-ANN in all evaluations except 2000. For DS4, IPA-ANN converged faster than BA-ANN and GOA-ANN in all evaluations. GA-ANN and FA-ANN had the best convergence. For DS5, IPA-ANN had the same convergence value as WOA-ANN at 2000 evaluations and provided worse convergence at 4000 evaluations. IPA-ANN converged better than all other algorithms. For DS6, IPA-ANN converged better than FA-ANN and WOA-ANN, except for 2000 and 4000 evaluations. It converged better than BA-ANN and GOA-ANN in all evaluations. GA-ANN provided the best convergence when all evaluations were examined. For DS7, IPA-ANN converged faster than all other algorithms. Only the 2000 evaluation part in FA-ANN converged better than IPA-ANN. For DS8, IPA-ANN provided faster convergence than all other algorithms. For DS9, it converged faster than all evaluations except for the 2000 evaluations in IPA-ANN, FA-ANN, GA-ANN, and WOA-ANN. It converged faster than all evaluations in IPA-ANN, BA-ANN, and GOA-ANN.

The experimental findings suggest that the effectiveness of IPA-ANN is influenced by the structural characteristics of the datasets and the complexity of the corresponding optimization search space. In datasets with relatively large feature spaces and more complex class distributions, such as DS1 and DS7, IPA-ANN demonstrated stable, competitive optimization behavior. This may be attributed to the donor–receiver interaction mechanism of IPA, which helps maintain population diversity while balancing exploration and exploitation during ANN weight optimization. On the other hand, in some datasets, such as DS3, DS5, DS8, and DS9, the performance differences between IPA-ANN and several competing metaheuristic approaches were limited. These datasets generally contain lower-dimensional structures, fewer classes, or comparatively simpler decision boundaries, reducing the relative advantage of advanced population interaction mechanisms. In such cases, multiple metaheuristic optimizers may converge to similar solution regions, leading to comparable classification performance.

Furthermore, the results indicate that IPA-ANN tends to provide more stable convergence behavior in problems associated with larger or more irregular optimization landscapes, whereas its relative superiority becomes less pronounced in simpler classification scenarios with smaller search spaces. These findings suggest that the effectiveness of IPA-based ANN optimization is closely related to the structural complexity and optimization difficulty of the classification problem.

The obtained findings are also generally consistent with previous ANN–metaheuristic hybrid studies reported in the literature. Similar to PSO-ANN-, GWO-ANN-, WOA-ANN-, and COOT-ANN-based models, the proposed IPA-ANN framework demonstrated that population-based optimization strategies can improve ANN training stability and reduce the risk of premature convergence by enhancing global search capability. However, as also reported in several previous hybrid ANN studies, the superiority of a specific metaheuristic optimizer is often problem-dependent and may vary according to dataset structure, optimization difficulty, and ANN search-space characteristics. Therefore, the findings of this study support the broader literature indicating that no single metaheuristic approach consistently dominates all classification scenarios.

### 5.1. Comparison with Conventional Gradient-Based ANN Training Baselines

While the primary scope of this study, as outlined in the Introduction, was intentionally restricted to a comparison among population-based, derivative-free metaheuristic optimizers, it is also important to contextualize the practical benefit of the proposed IPA-ANN framework relative to conventional gradient-based ANN training. To this end, a supplementary experiment was conducted in which the same fixed ANN architecture described in Section 3.3 (two hidden layers with 10 and 8 neurons, ReLU activations, a Softmax output layer, and a categorical cross-entropy loss function) was trained using five widely adopted gradient-based optimizers: Stochastic Gradient Descent (SGD), SGD with momentum, RMSprop, Adam, and Adagrad. Consistent with the representative-dataset approach adopted in Section 5.3, this analysis was performed on two datasets selected to reflect contrasting problem scales: DS7, the smallest dataset used in this study, and DS2, the largest dataset in terms of sample size and feature dimensionality. For both datasets, an identical fixed 80/20 train–test partition was employed, matching the evaluation protocol used for the metaheuristic–ANN comparisons in Section 5. Each optimizer was evaluated over 10 independent runs, with only the network’s initial weights and mini-batch ordering varying between runs, and the resulting mean, standard deviation, best, and worst test-accuracy values were recorded. Table 6 presents the results of this comparison.

Table 6 presents the test accuracy of the five gradient-based optimizers on DS2 and DS7. A pronounced contrast emerges between the two datasets. On DS2, all five gradient-based optimizers achieve mean test accuracies between 0.9825 and 0.9920, substantially exceeding the best mean accuracy obtained by any metaheuristic-based ANN model reported in Table 3, namely GWO-ANN (0.8150). This gap of approximately 17–18 percentage points indicates that, for this comparatively large and high-dimensional dataset, gradient-based backpropagation is markedly more effective at navigating the ANN weight space than population-based, derivative-free search under an equal evaluation budget.

On DS7, however, this advantage largely disappears. The mean accuracies of the gradient-based optimizers (0.9467–0.9700) are comparable to, and in some cases lower than, the best-performing metaheuristic configurations reported for this dataset: IPA_3_3-ANN achieved a mean accuracy of 0.9667 under single-fold evaluation (Table 5), matching SGD and RMSprop and exceeding SGD-Momentum (0.9467) and Adam (0.9600), while GBO-ANN, MRFO-ANN, and PSO-ANN achieved higher mean accuracies (0.9854, 0.9840, and 0.9813, respectively) under the repeated cross-validation protocol reported in Section 5.3.2. This pattern suggests that the practical benefit of gradient-based training over population-based metaheuristic optimization is closely tied to dataset scale and feature dimensionality: with only 120 training samples and 4 features, the limited amount of gradient information available per epoch reduces the relative efficiency advantage of backpropagation, allowing global population-based search to remain competitive.

It should be noted that this comparison, while informative for practical contextualization, is not fully commensurable in a strict methodological sense. Gradient-based optimizers exploit analytical derivative information and were trained for a fixed number of epochs and mini-batch updates, whereas the metaheuristic-based models in this study are entirely derivative-free and were constrained to a fixed number of fitness evaluations; these two computational budgets are not directly equivalent. Furthermore, gradient-based training benefits from a differentiable, smoothly navigable loss landscape, a property that population-based metaheuristics do not require and that constitutes one of their principal motivations in problems with non-differentiable, discontinuous, or highly multimodal objective functions. Accordingly, the results in Table 6 should be interpreted as practical context on the relative behavior of the two optimization paradigms under comparable architectural and data-partitioning conditions, rather than as a strictly fair, budget-matched benchmark between fundamentally different classes of optimization algorithms.

### 5.2. Statistical Validation Across All Nine Datasets via Friedman Test

To complement the dataset-specific validation reported in Section 5.3, a statistical assessment of the observed performance differences was additionally conducted across all nine datasets used in this study (DS1–DS9). Rather than restricting the analysis to a representative subset, this evaluation examines whether the differences in mean test accuracy among the 22 compared algorithm configurations—the 18 competing metaheuristic-based ANN models together with the four IPA-ANN donor–receiver variants—are statistically significant when considered jointly across the full set of benchmark datasets.

For this purpose, the non-parametric Friedman test was employed, which is widely recognized as the standard procedure for comparing multiple algorithms over multiple datasets [47]. The Friedman test evaluates the null hypothesis that all compared algorithms achieve equal average performance across datasets by ranking each algorithm within each dataset and analyzing the resulting distribution of ranks, thereby avoiding the distributional assumptions required by parametric alternatives such as repeated-measures ANOVA. The test was applied to the mean test accuracy values already reported for all nine datasets in Table 3, Table 4 and Table 5 and was followed by a post hoc pairwise Wilcoxon signed-rank analysis, with Holm–Bonferroni correction applied across the resulting simultaneous comparisons, between the best-performing IPA-ANN configuration and each of the remaining 21 algorithms.

The Friedman test applied across all nine datasets yielded $\chi^{2}=44.279$ (df=21, p=0.0022), rejecting the null hypothesis of equal performance among the 22 compared algorithm configurations at α=0.05 and thereby confirming that the observed differences in classification accuracy are statistically significant when evaluated jointly across DS1–DS9. As reported in Table 7, GWO-ANN obtained the best average rank (5.000), followed by DE-ANN (6.167) and IPA_3_3-ANN (7.778), which ranked third among the 22 configurations and was the best-performing among the four IPA-ANN donor–receiver variants, consistent with the per-dataset rankings reported in Table 3, Table 4 and Table 5. It should be noted that this ranking differs methodologically from the best-value ranking discussed at the end of Section 5, which selects, for each dataset, the best-performing among the four IPA-ANN donor–receiver variants and treats it as a single combined entry. The Friedman ranking presented here instead evaluates each of the four IPA-ANN configurations independently, using mean rather than best test accuracy, alongside the 18 competing algorithms under identical conditions, and therefore constitutes a more conservative and statistically rigorous assessment. The subsequent post hoc analysis (Table 8) shows that IPA_3_3-ANN achieved a higher mean accuracy than 20 of the 21 compared algorithms, being outperformed only by GWO-ANN; however, none of the individual pairwise differences survived the Holm–Bonferroni correction, a consequence of the limited statistical power inherent to a nine-sample paired test under correction for 21 simultaneous comparisons rather than an indication that no genuine difference exists, as already established by the significant omnibus Friedman result. Taken together, these results indicate that IPA-ANN, in its best-performing configuration, ranks among the top three of the 22 evaluated algorithms and that the overall performance differences across the nine benchmark datasets are statistically significant, providing dataset-wide support for the classification performance trends reported throughout the manuscript.

### 5.3. Validation via Repeated Stratified 5-Fold Cross-Validation on Small Datasets

To further examine the robustness of the reported single-fold results on datasets with limited sample sizes, a repeated stratified 5-fold cross-validation analysis (5 repetitions) was additionally conducted for the DS6 and Iris DS7 datasets, which were selected given the two were the smallest datasets used in this study. For each dataset, the IPA-ANN model was evaluated across 25 folds in total (5 folds × 5 repetitions), and the resulting test accuracy values were analyzed from three complementary perspectives. First, descriptive statistics (mean, standard deviation, median, first and third quartiles, minimum, maximum, and interquartile range) were computed to characterize the central tendency and dispersion of the cross-validated accuracy distribution. Second, boxplot visualizations were generated to provide a graphical comparison between the single-fold and cross-validated results. Finally, statistical significance tests were performed to assess whether the observed differences between the single-fold and cross-validated accuracy distributions are statistically meaningful. The results of this three-part analysis are presented in the following subsections.

#### 5.3.1. Detailed Results for DS6

Table 9 reports the descriptive statistics of the test accuracy obtained via repeated stratified 5-fold cross-validation on the DS6 dataset. Among all 22 compared algorithms, PSO-ANN and BBO-ANN achieved the highest mean accuracy (0.8374), followed by GBO-ANN (0.8310) and GWO-ANN (0.8287). Among the four IPA-ANN configurations, IPA_1_2-ANN obtained the highest mean accuracy (0.8167), followed by IPA_4_4-ANN (0.8154), IPA_3_3-ANN (0.8129), and IPA_2_2-ANN (0.8090). Notably, this ranking differs from the single-fold results reported in Table 4, where IPA_3_3-ANN achieved the highest average test accuracy (0.8258) among all compared algorithms on DS6. This discrepancy indicates that mean performance rankings obtained from a single 80/20 split can vary across evaluation protocols, particularly on small datasets such as Hepatitis, and underlines the importance of complementing single-fold evaluations with cross-validation to obtain a more comprehensive assessment of generalization performance.

In terms of stability, IPA_2_2-ANN exhibited the lowest standard deviation (0.0032) and the narrowest interquartile range (IQR = 0.0065) among all 22 algorithms, closely followed by DE-ANN (Std = 0.0045) and TLBO-ANN (Std = 0.0065). This indicates that, although IPA_2_2-ANN did not attain the highest mean accuracy, it produced the most consistent classification performance across folds and repetitions, a desirable property for reliable deployment on small, high-variance datasets. In contrast, PSO-ANN, despite achieving the highest mean accuracy, exhibited the largest standard deviation (0.0327) and the widest IQR (0.0637) among all algorithms, suggesting comparatively less stable behavior across folds. Overall, while the IPA-ANN configurations do not dominate the ranking in terms of mean accuracy under the 5-fold cross-validation protocol on this dataset, they demonstrate competitive and, in the case of IPA_2_2-ANN, notably stable performance relative to the majority of the compared metaheuristic algorithms.

**Figure 21:**
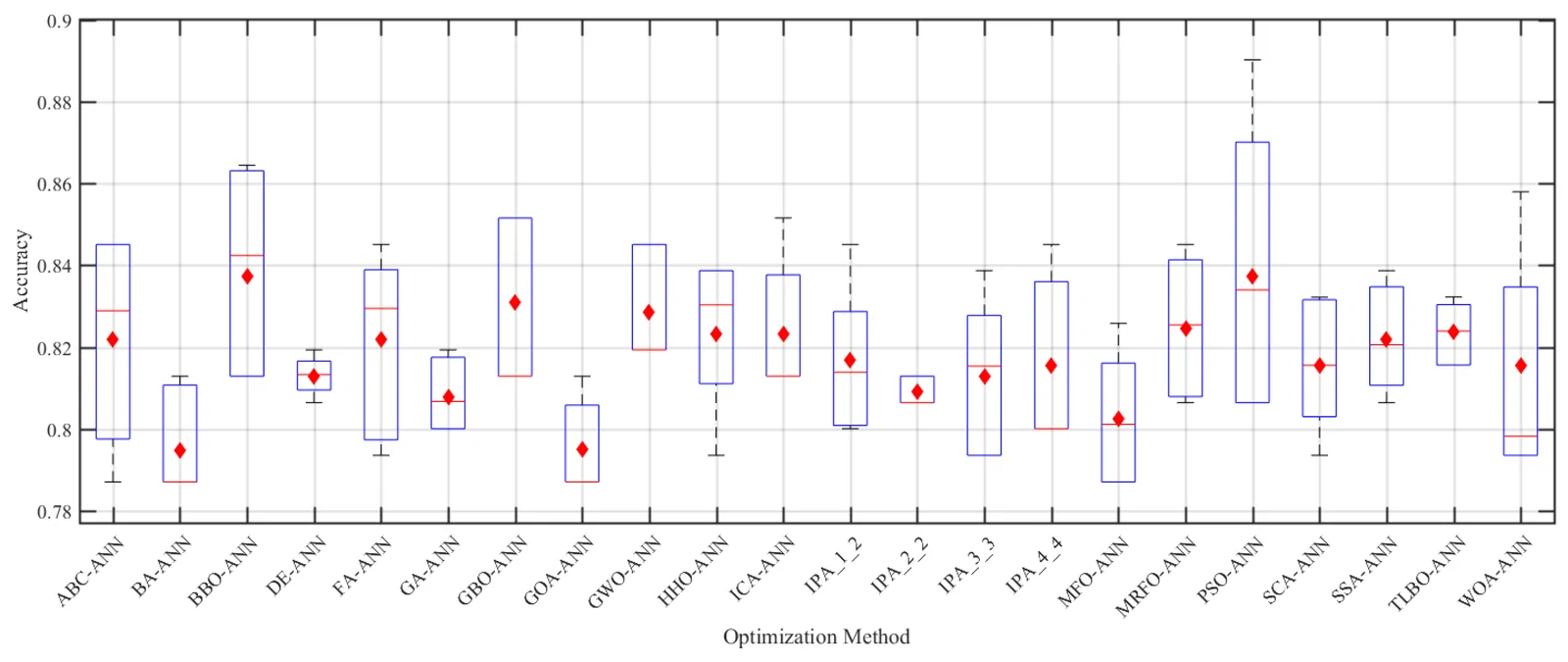
Boxplot distribution of test accuracy values obtained via repeated stratified 5-fold cross-validation (5 repetitions, 25 folds in total) for the compared algorithms on the DS6 dataset. The red horizontal line indicates the median, and the red diamond marker indicates the mean accuracy for each algorithm.

Figure 21 presents the boxplot distributions of the test accuracy values obtained via 5-fold cross-validation for all compared algorithms on the DS6 dataset, complementing the descriptive statistics reported in Table 9. The results confirm that IPA_2_2-ANN exhibits the narrowest interquartile range among all algorithms, with its box compressed into a visibly tight band and minimal separation between the median and mean markers, reflecting the low variance and consistent behavior already indicated by its standard deviation value. A similarly compact distribution is observed for DE-ANN, whereas PSO-ANN, despite achieving the highest mean accuracy, displays one of the widest boxes and the largest whisker span in the figure, extending from approximately 0.806 to 0.890, which visually corroborates its comparatively high standard deviation and IQR reported in Table 9. Several algorithms, including BBO-ANN, GBO-ANN, and WOA-ANN, also show relatively wide interquartile ranges, indicating higher sensitivity to the specific fold composition. Among the IPA-ANN configurations, IPA_1_2-ANN, IPA_3_3-ANN, and IPA_4_4-ANN display moderate dispersion comparable to several competing algorithms such as HHO-ANN and ICA-ANN, whereas IPA_2_2-ANN stands out as the most stable configuration overall. Taken together, the boxplot analysis reinforces the observation drawn from the descriptive statistics: on the DS6 dataset, competitive mean accuracy and low variance are not necessarily achieved simultaneously by the same algorithm, and the IPA-ANN framework, particularly through the IPA_2_2 configuration, offers a favorable trade-off in terms of prediction stability under repeated cross-validation.

To statistically assess whether the observed differences in test accuracy between the proposed IPA-ANN model and the compared algorithms are significant, the paired Wilcoxon signed-rank test was applied to the 25 cross-validation accuracy values (5 repetitions × 5 folds) obtained for each algorithm on the DS6 dataset. Among the four IPA-ANN configurations, the variant achieving the highest mean cross-validated accuracy, namely IPA_1_2-ANN, was selected as the representative proposed method and compared against each of the remaining 21 algorithms individually. Since this procedure involves 21 pairwise comparisons conducted simultaneously, the Holm–Bonferroni correction was applied to the resulting *p*-values to control the family-wise error rate and reduce the likelihood of Type I errors. For each comparison, a corrected *p*-value below the significance threshold (α=0.05) was considered to indicate a statistically significant difference between the two algorithms, with the winning method determined according to the higher mean accuracy.

Table 10 presents the pairwise Wilcoxon signed-rank test results, together with the Holm–Bonferroni correction, between IPA_1_2-ANN and the remaining 21 algorithms on the DS6 dataset. Based on mean accuracy, IPA_1_2-ANN outperformed 10 of the 21 compared algorithms while being outperformed by the remaining 11, indicating a balanced overall performance profile rather than a clear dominance. However, after applying the Holm–Bonferroni correction, only three of the 21 pairwise comparisons reached statistical significance at the α=0.05 level: IPA_1_2-ANN was found to be significantly superior to BA-ANN (adjusted p=0.0019) and GOA-ANN (adjusted p=0.0025), whereas BBO-ANN was found to be significantly superior to IPA_1_2-ANN (adjusted p=0.0041). For all remaining comparisons, including those against several algorithms with higher mean accuracy such as GBO-ANN, GWO-ANN, and PSO-ANN, the corrected *p*-values exceeded the significance threshold, indicating that the observed differences in mean accuracy are not statistically significant and may be attributable to the variability inherent in a small dataset such as Hepatitis. These results suggest that, on the DS6 dataset, IPA_1_2-ANN achieves a level of classification performance that is statistically indistinguishable from the majority of the compared metaheuristic algorithms, while demonstrating a statistically significant advantage over a subset of them.

#### 5.3.2. Detailed Results for DS7

Table 11 reports the descriptive statistics of the test accuracy obtained via repeated stratified 5-fold cross-validation on the DS7 dataset. Among all 22 compared algorithms, GBO-ANN achieved the highest mean accuracy (0.9854), followed closely by MRFO-ANN (0.9840) and PSO-ANN (0.9813). Among the four IPA-ANN configurations, IPA_2_2-ANN clearly outperformed the remaining variants, obtaining a mean accuracy of 0.9692, which ranks eighth among all 22 algorithms and is comparable to well-established methods such as TLBO-ANN (0.9720) and DE-ANN (0.9667). In contrast, the other three IPA-ANN configurations—namely IPA_4_4-ANN (0.9546), IPA_3_3-ANN (0.9318), and particularly IPA_1_2-ANN (0.8440)—exhibited noticeably lower mean accuracy on this dataset, with IPA_1_2-ANN ranking among the lowest-performing configurations overall. This is consistent with the single-fold results discussed in Table 5, where the donor–receiver parameter setting was shown to have a considerable effect on convergence and accuracy depending on the dataset, further reinforcing the observation that no single IPA-ANN configuration is uniformly optimal across all datasets.

In terms of stability, IPA_2_2-ANN also maintained a relatively low standard deviation (0.0165) and a narrow interquartile range (IQR = 0.0237), ranking among the more consistent algorithms alongside SCA-ANN (Std = 0.0033), DE-ANN (Std = 0.0124), and MRFO-ANN (Std = 0.0129). Conversely, IPA_1_2-ANN displayed both a low mean accuracy and a comparatively high standard deviation (0.0816), indicating unstable performance across folds for this particular dataset. Several non-IPA algorithms, including GWO-ANN (Std = 0.1560), GOA-ANN (Std = 0.1550), and MFO-ANN (Std = 0.1430), exhibited even greater variability, despite GWO-ANN attaining a moderately high mean accuracy, suggesting a tendency toward inconsistent convergence across folds. Overall, the cross-validation results on the Iris dataset indicate that the IPA_2_2-ANN configuration provides the most reliable trade-off between accuracy and stability among the proposed variants, whereas the remaining IPA-ANN configurations, in particular IPA_1_2-ANN, underperform relative to several competing algorithms on this dataset.

**Figure 22:**
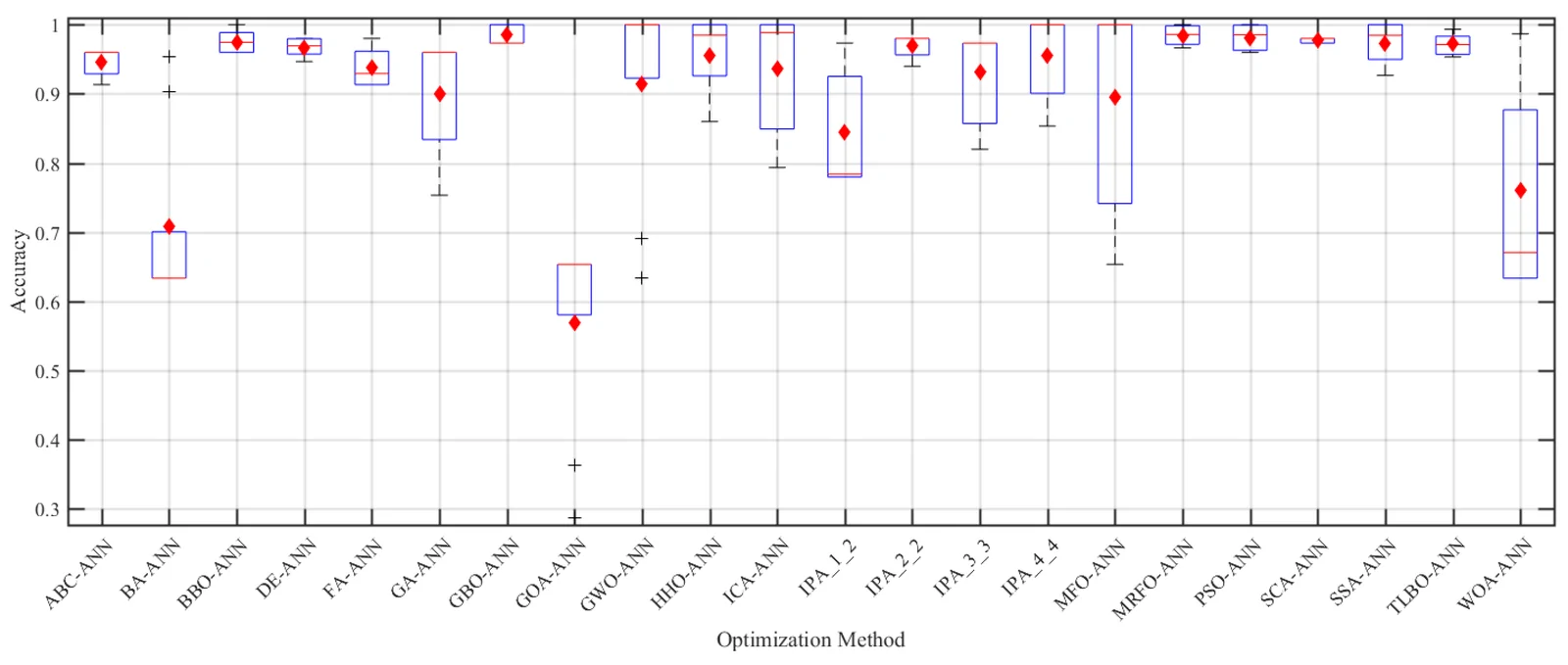
Boxplot distribution of test accuracy values obtained via repeated stratified 5-fold cross-validation (5 repetitions, 25 folds in total) for the compared algorithms on the DS7 dataset. The red horizontal line indicates the median, and the red diamond marker indicates the mean accuracy for each algorithm.

Figure 22 presents the boxplot distributions of the test accuracy values obtained via 5-fold cross-validation for all compared algorithms on the DS7 dataset, complementing the descriptive statistics reported in Table 11. Among the IPA-ANN configurations, IPA_2_2-ANN exhibits a compact box concentrated near the upper end of the accuracy range, with minimal separation between its median and mean markers, visually confirming the low variance and high mean accuracy already reported in Table 11. In contrast, IPA_1_2-ANN displays the widest and lowest-positioned box among the four IPA variants, spanning from approximately 0.780 to 0.925 with a mean accuracy noticeably below its median, indicating both reduced accuracy and higher fold-to-fold variability. IPA_3_3-ANN and IPA_4_4-ANN show intermediate behavior, with wider interquartile ranges than IPA_2_2-ANN but narrower whisker spans than IPA_1_2-ANN. Among the competing algorithms, GOA-ANN, BA-ANN, and WOA-ANN exhibit markedly lower and more dispersed distributions, with GOA-ANN additionally showing several outliers below 0.4, reflecting unstable convergence behavior on this dataset. Conversely, GBO-ANN, MRFO-ANN, PSO-ANN, and SCA-ANN display compact boxes tightly clustered near the maximum accuracy value, consistent with their top-ranking mean accuracy and low standard deviation values in Table 11. Overall, the boxplot analysis reinforces the conclusion that IPA_2_2-ANN provides the most favorable balance between accuracy and consistency among the IPA-ANN configurations on the DS7 dataset, whereas IPA_1_2-ANN is comparatively less reliable in this setting.

Table 12 presents the pairwise Wilcoxon signed-rank test results, together with the Holm–Bonferroni correction, between IPA_2_2-ANN and the remaining 21 algorithms on the DS7 dataset. Based on mean accuracy, IPA_2_2-ANN outperformed 14 of the 21 compared algorithms while being outperformed by only 7, indicating a favorable overall performance profile on this dataset. After applying the Holm–Bonferroni correction, 10 of the 21 pairwise comparisons reached statistical significance at the α=0.05 level. Specifically, IPA_2_2-ANN was found to be significantly superior to ABC-ANN, BA-ANN, FA-ANN, GA-ANN, GOA-ANN, IPA_1_2-ANN, IPA_3_3-ANN, and WOA-ANN, whereas GBO-ANN and MRFO-ANN were found to be significantly superior to IPA_2_2-ANN. Notably, the significant advantage of IPA_2_2-ANN over IPA_1_2-ANN (adjusted p=0.0008) and IPA_3_3-ANN (adjusted p=0.0284) confirms that the choice of donor–receiver parameters within the IPA-ANN framework has a statistically meaningful effect on classification performance for this dataset. For the remaining comparisons, including those against BBO-ANN, PSO-ANN, and SCA-ANN, which achieved slightly higher mean accuracy, the corrected *p*-values did not reach significance, indicating that the observed differences are not statistically distinguishable from IPA_2_2-ANN’s performance. Taken together, these results indicate that IPA_2_2-ANN provides a statistically competitive, and in several cases significantly superior, classification performance relative to the majority of the compared metaheuristic algorithms on the DS7 dataset.

Overall, the repeated stratified 5-fold cross-validation analysis conducted on the two smallest datasets, DS6 and DS7, indicates that the reported single-fold results are broadly consistent with the cross-validated performance of the IPA-ANN framework, while also revealing that the choice of donor–receiver parameters can have a dataset-dependent and, in some cases, statistically significant effect on classification accuracy. On DS6, the best-performing IPA-ANN configuration (IPA_1_2-ANN) achieved a balanced performance profile with no dominant advantage over the majority of the compared algorithms, whereas on DS7, IPA_2_2-ANN demonstrated a statistically superior performance over a considerable proportion of the compared methods, including the other IPA-ANN variants. These findings collectively support the robustness of the proposed model under a more rigorous validation protocol, while also confirming that the selection of the donor–receiver parameters remains an important factor influencing the performance of the IPA-ANN framework across different datasets.

### 5.4. Computational Cost Comparison on the DS7

To address the computational cost of the compared algorithms, the CPU execution time required for each of the 10,000 fitness evaluations was recorded for all 22 algorithm configurations (18 competing algorithms and 4 IPA-ANN variants) during a single training run on the DS7 dataset. All algorithms were executed within the same run and under identical hardware and software conditions, ensuring that the reported timing differences reflect the relative computational overhead of each algorithm’s update mechanism rather than variations in the execution environment. It should be noted that the reported values correspond to a single representative run of 10,000 evaluations rather than an average over multiple repetitions, and are therefore intended to provide a practical indication of relative computational cost rather than a statistically exhaustive runtime analysis.

Table 13 reports the mean per-evaluation CPU time and the total CPU time over 10,000 evaluations for all 22 compared algorithm configurations on the Iris dataset. The results indicate that the vast majority of the algorithms exhibit remarkably similar computational overhead, with mean per-evaluation times ranging narrowly between approximately 0.310 s (PSO-ANN) and 0.321 s (GOA-ANN), corresponding to total execution times between roughly 51.7 and 53.5 min. This narrow range indicates that, for the majority of the compared algorithms, the dominant computational cost is attributable to the repeated ANN fitness evaluation (i.e., the forward pass and cross-entropy loss computation) rather than to the internal update mechanism of the metaheuristic itself, since all algorithms share the same population size (PS = 30) and the same number of fitness evaluations (10,000). The four IPA-ANN variants (IPA_1_2-ANN, IPA_2_2-ANN, IPA_3_3-ANN, and IPA_4_4-ANN) fall within this narrow band, with total execution times ranging from approximately 52.16 to 52.58 min, indicating that the donor–receiver mechanism introduces negligible additional computational overhead compared to the other population-based metaheuristics evaluated. The only notable exception is FA-ANN, which exhibits both a higher mean per-evaluation time (0.3664 s) and a substantially larger standard deviation (0.2608 s) compared to all other algorithms, resulting in a total execution time of approximately 61.07 min, roughly 15–18% longer than the remaining algorithms. This increased and more variable computational cost is consistent with the pairwise attractiveness-based update mechanism of the FA, which requires computing distances and attractiveness values between multiple individual pairs at each evaluation, in contrast to the comparatively simpler update rules employed by the other compared algorithms. Overall, these results indicate that the proposed IPA-ANN framework achieves its reported classification performance without incurring additional computational cost relative to the majority of the competing metaheuristic algorithms.

## 6. Conclusions and Future Work

The proposed IPA-ANN model in this study was compared with 18 metaheuristic algorithms across 9 datasets. Evaluations based on average and best test-accuracy values, confusion matrices, and convergence graphs yielded the following findings:

When examining IPA-ANN combinations for single-fold validation across all datasets, IPA_3_3-ANN and IPA_4_4-ANN mostly achieved the best accuracy in terms of average test accuracy values. In terms of best values, IPA_2_2-ANN and IPA_3_3-ANN achieved the highest accuracies. In the best-value section, IPA-ANN combinations outperformed other algorithms in most datasets. Overall, IPA-ANN combinations performed better than other algorithms, with higher average and best test accuracies.

When confusion matrices were examined, it was observed that the IPA-ANN model achieved higher classification accuracy than most competing algorithms. The IPA combinations used in the study generally resulted in fewer misclassifications. These findings show that the proposed model achieves high classification accuracy across most datasets.

It was observed that changing the donor–receiver parameters of IPA had an effect on convergence performance. While IPA_1_2-ANN generally provided fast convergence at 2000 and 4000 evaluation counts, IPA_3_3-ANN and IPA_4_4-ANN achieved higher test accuracy in some datasets. In other datasets, the optimal convergence parameter varied depending on the dataset. The results obtained show that IPA-ANN offers variable values in terms of test accuracy and convergence performance depending on the dataset. This should be regarded as a preliminary observation derived from the convergence trends within a single fixed evaluation budget, rather than a validated recommendation across evaluation budgets, since the donor and receiver individuals are updated adaptively as the number of evaluations approaches the maximum evaluation count, and this behavior may therefore differ under independently defined, reduced evaluation budgets. Confirming and generalizing this observation would require dedicated experiments conducted under a range of independently defined evaluation budgets, which is identified as a direction for future work. This finding highlights that the donor–receiver parameters of IPA should be selected according to the problem at hand.

A supplementary comparison with five conventional gradient-based ANN training methods (SGD, SGD with momentum, RMSprop, Adam, and Adagrad) was additionally conducted on two representative datasets to contextualize the practical benefit of the proposed framework. The results revealed a dataset-dependent pattern: gradient-based optimizers achieved substantially higher accuracy on the large-scale DS2 dataset, whereas on the small-scale DS7 dataset, their performance was comparable to, and in some cases lower than, the best-performing metaheuristic–ANN configurations. Furthermore, a Friedman test applied across all nine datasets confirmed that the observed performance differences among the 22 compared algorithm configurations are statistically significant ($\chi^{2}=44.279$, df=21, p=0.0022), with IPA_3_3-ANN ranking third overall among all evaluated configurations.

To further assess the reliability of the single-fold results and to statistically substantiate the reported performance differences, a supplementary evaluation was conducted using repeated stratified 5-fold cross-validation on the two smallest datasets, Hepatitis and Iris, together with paired Wilcoxon signed-rank tests and Holm–Bonferroni correction between the best-performing IPA-ANN configuration and each of the remaining 21 algorithms. The cross-validated results were found to be broadly consistent with the single-fold findings, while also confirming that a subset of the observed performance differences, including several in favor of IPA-ANN, are statistically significant rather than attributable to random variation. In addition, a computational cost analysis conducted on the Iris dataset showed that the IPA-ANN configurations incur computational overhead comparable to that of the majority of the compared algorithms, indicating that the reported classification performance is not achieved at the expense of additional computational cost.

In general, the IPA-ANN algorithm has provided higher test accuracy, lower misclassification rates, and, in several cases, faster convergence across most datasets compared to the 18 different optimization algorithms evaluated, without incurring additional computational cost. Furthermore, it has been observed that the algorithm delivers robust and reliable classification performance when its donor–receiver parameters are appropriately selected for the problem at hand.

Although the proposed IPA-ANN framework demonstrated competitive performance across multiple classification tasks, several limitations should be noted. The experimental analysis primarily relied on a single train–test partition with 10 independent runs across all nine datasets; while this concern was partially addressed through a supplementary repeated stratified 5-fold cross-validation analysis on two representative small datasets, extending this validation protocol to all datasets remains an important direction for future work. In addition, a fixed ANN architecture was employed for all datasets to ensure methodological consistency and fair comparison among optimization algorithms; however, dataset-specific architectures and adaptive hyperparameter tuning may further improve performance. Moreover, the relative advantage of IPA-ANN was not uniformly observed across all datasets, particularly in problems characterized by simpler optimization landscapes.

Future research may focus on cross-validation-driven evaluation across all benchmark datasets, adaptive ANN design, larger and more diverse benchmark datasets, and hybrid or enhanced IPA-based optimization strategies. Also, a redesign of the ANN structure used in the model is planned, incorporating various activation functions, layer numbers, and learning strategies. Furthermore, creating an improved version of the proposed model and designing a better fitness function to reanalyze its performance on various datasets are among ideas for future studies. In this context, integrating alternative optimization strategies to improve the model’s prediction accuracy is considered a future research topic.

## Figures and Tables

**Table 1:** Properties of datasets.

Dataset	Feature	Instances	Class	Class Labels
Breast Cancer Wisconsin	9	699	2	benign, malignant
Chess (King-Rook vs. King-Pawn)	35	3196	2	won → white won, nowin → white lost
Credit Approval	15	690	2	+ → credit is approved, − → credit denied
Early-Stage Diabetes Risk Prediction	16	520	2	positive → there is a risk of diabetes, negative → no risk of diabetes
Heart Disease	13	303	5	no disease, mild disease, moderate disease, serious disease, very serious disease
Hepatitis	19	155	2	living, deceased
Iris	4	150	3	iris-setosa, iris-versicolor, iris-virginica
Tic-Tac-Toe Endgame	9	958	2	positive → X is winning, negative → X cannot win
Congressional Voting Records	16	435	2	Democrat, Republican

**Table 2:** Abbreviations of the datasets.

Dataset Abbreviation	Dataset Name
DS1	Breast Cancer
DS2	Chess
DS3	Credit Approval
DS4	Diabetes
DS5	Heart Disease
DS6	Hepatitis
DS7	Iris
DS8	Tic-Tac-Toe
DS9	Voting Records

**Table 3:** Algorithms’ DS1–DS3 test accuracy and F1-score values for a single fold.

Algorithm	DS1					DS2					DS3				
	Mean	Std	Best	Worst	F1-Score	Mean	Std	Best	Worst	F1-Score	Mean	Std	Best	Worst	F1-Score
ABC-ANN	0.9171	0.0160	0.9429	0.9000	0.9431	0.7922	0.0088	0.8047	0.7812	0.7919	0.5841	0.0074	0.5942	0.5725	0.4623
BA-ANN	0.7971	0.0615	0.9071	0.7357	0.9090	0.7569	0.0050	0.7641	0.7484	0.6693	0.5870	0.0000	0.5870	0.5870	0.4342
BBO-ANN	0.9329	0.0107	0.9429	0.9143	0.9429	0.8016	0.0065	0.8141	0.7953	0.7930	0.5783	0.0029	0.5797	0.5725	0.4308
DE-ANN	0.9400	0.0107	0.9500	0.9214	0.9501	0.8053	0.0095	0.8188	0.7891	0.8017	0.5870	0.0079	**0.6014**	0.5797	0.4662
FA-ANN	0.8829	0.0195	0.9071	0.8571	0.9046	0.7741	0.0140	0.7891	0.7500	0.7547	0.5841	0.0035	0.5870	0.5797	0.4342
GA-ANN	0.9071	0.0256	0.9429	0.8714	0.9435	0.7781	0.0113	0.7984	0.7656	0.7838	0.5884	0.0071	**0.6014**	0.5797	0.4662
GBO-ANN	0.9400	0.0107	0.9500	0.9214	0.9503	0.7969	0.0100	0.8062	0.7812	0.7911	0.5797	0.0079	0.5870	0.5620	0.4342
GOA-ANN	0.8771	0.0379	0.9214	0.8214	0.9222	0.7563	0.0000	0.7563	0.7563	0.6513	0.5870	0.0000	0.5870	0.5870	0.4342
GWO-ANN	0.9486	0.0053	**0.9571**	0.9429	0.9573	0.8150	0.0036	**0.8203**	0.8094	**0.8162**	0.5739	0.0174	0.5942	0.5435	0.4623
HHO-ANN	0.9357	0.0057	0.9500	0.9257	0.9505	0.7772	0.0140	0.8031	0.7641	0.7999	0.5826	0.0118	0.5942	0.5652	0.4504
ICA-ANN	0.8943	0.0528	0.9500	0.9286	0.9503	0.7722	0.0123	0.7953	0.7594	0.7560	0.5710	0.0186	0.5870	0.5362	0.4772
IPA_1_2-ANN	0.9243	0.0160	0.9500	0.9000	0.9503	0.7812	0.0062	0.7922	0.7734	0.7747	0.5899	0.0058	**0.6014**	0.5870	**0.4774**
IPA_2_2-ANN	0.9400	0.0073	0.9500	0.9286	0.9501	0.7809	0.0084	0.7906	0.7672	0.7877	0.5797	0.0065	0.5870	0.5725	0.4342
IPA_3_3-ANN	0.9443	0.0070	**0.9571**	0.9357	**0.9575**	0.8009	0.0118	0.8141	0.7828	0.8056	0.5899	0.0074	**0.6014**	0.5797	0.4662
IPA_4_4-ANN	0.9443	0.0053	0.9500	0.9357	0.9503	0.7800	0.0052	0.7859	0.7719	0.7636	0.5768	0.0098	0.5870	0.5652	0.4342
MFO-ANN	0.9371	0.0131	0.9500	0.9214	0.9503	0.7669	0.0158	0.7969	0.7563	0.7585	0.5870	0.0046	0.5942	0.5797	0.4623
MRFO-ANN	0.9400	0.0107	0.9500	0.9214	0.9503	0.7825	0.0164	0.8078	0.7578	0.7945	0.5913	0.0074	**0.6014**	0.5797	0.4662
PSO-ANN	0.9257	0.0291	0.9500	0.8714	0.9501	0.8009	0.0096	0.8141	0.7859	0.8019	0.5884	0.0029	0.5942	0.5870	0.4623
SCA-ANN	0.9329	0.0160	0.9500	0.9143	0.9505	0.8016	0.0092	0.8141	0.7859	0.7977	0.5870	0.0046	0.5942	0.5797	0.4504
SSA-ANN	0.9243	0.0147	0.9357	0.9000	0.9356	0.7956	0.0149	0.8094	0.7719	0.7899	0.5812	0.0085	0.5870	0.5652	0.4468
TLBO-ANN	0.9414	0.0070	0.9500	0.9286	0.9503	0.8056	0.0060	0.8125	0.7984	0.8030	0.5841	0.0035	0.5870	0.5797	0.4342
WOA-ANN	0.8571	0.0517	0.9214	0.8000	0.9215	0.7594	0.0085	0.7703	0.7484	0.7203	0.5870	0.0046	0.5942	0.5797	0.4733
**IPA-ANN ranking**			1		1			3		2			1		1

**Table 4:** Algorithms’ DS4–DS6 test accuracy and F1-score values for a single fold.

Algorithm	DS4					DS5					DS6				
	Mean	Std	Best	Worst	F1-Score	Mean	Std	Best	Worst	F1-Score	Mean	Std	Best	Worst	F1-Score
ABC-ANN	0.8769	0.0112	0.8846	0.8558	0.8305	0.6623	0.0080	0.6721	0.6557	0.5653	0.7677	0.0474	0.8387	0.7097	0.7884
BA-ANN	0.8846	0.0000	0.8846	0.8846	0.8305	0.6230	0.0180	0.6557	0.6066	0.5509	0.7677	0.0801	0.8387	0.6129	0.7884
BBO-ANN	0.8808	0.0047	0.8846	0.8750	0.8305	0.6328	0.0167	0.6557	0.6066	0.5519	0.7806	0.0774	**0.8710**	0.6452	0.8435
DE-ANN	0.8808	0.0077	0.8846	0.8654	0.8464	0.6492	0.0222	0.6721	0.6066	0.5642	0.7871	0.0158	0.8065	0.7742	0.7200
FA-ANN	0.8788	0.0077	0.8846	0.8654	0.8305	0.6426	0.0123	0.6557	0.6230	0.5409	0.7742	0.0204	0.8065	0.7419	0.7200
GA-ANN	0.8827	0.0038	0.8846	0.8750	0.8305	0.6393	0.0180	0.6557	0.6066	0.5421	0.7871	0.0329	0.8387	0.7419	0.8164
GBO-ANN	0.8750	0.0172	0.8942	0.8462	0.8525	0.6459	0.0382	**0.6885**	0.5738	**0.6005**	0.7613	0.0752	0.8065	0.6129	0.7652
GOA-ANN	0.8846	0.0000	0.8846	0.8846	0.8305	0.6230	0.0000	0.6230	0.6230	0.4782	0.8065	0.0000	0.8065	0.8065	0.7200
GWO-ANN	0.8865	0.0094	**0.9038**	0.8750	**0.8818**	0.6492	0.0131	0.6557	0.6230	0.5421	0.7871	0.0438	0.8387	0.7097	0.7884
HHO-ANN	0.8827	0.0072	0.8942	0.8750	0.8525	0.6426	0.0161	0.6557	0.6230	0.5519	0.7484	0.0428	0.8065	0.6774	0.7200
ICA-ANN	0.8808	0.0077	0.8846	0.8654	0.8672	0.6426	0.0123	0.6557	0.6230	0.5421	0.7613	0.0598	0.8387	0.6774	0.8330
IPA_1_2-ANN	0.8885	0.0128	**0.9038**	0.8654	0.8720	0.6230	0.0180	0.6557	0.6066	0.5519	0.7419	0.0577	0.8065	0.6452	0.7200
IPA_2_2-ANN	0.8846	0.0000	0.8846	0.8846	0.8305	0.6361	0.0191	0.6721	0.6230	0.5642	0.7742	0.0289	0.8065	0.7419	0.7200
IPA_3_3-ANN	0.8808	0.0098	0.8942	0.8654	0.8525	0.6164	0.0338	0.6557	0.5574	0.5519	0.8258	0.0329	**0.8710**	0.7742	**0.8605**
IPA_4_4-ANN	0.8692	0.0198	0.8846	0.8365	0.8305	0.6459	0.0197	0.6721	0.6230	0.5573	0.7806	0.0474	0.8387	0.7097	0.7884
MFO-ANN	0.8846	0.0000	0.8846	0.8846	0.8305	0.6361	0.0161	0.6557	0.6230	0.5519	0.7806	0.0516	0.8065	0.6774	0.7200
MRFO-ANN	0.8769	0.0112	0.8846	0.8558	0.8305	0.6426	0.0161	0.6557	0.6230	0.5535	0.7613	0.0438	0.8065	0.7097	0.7652
PSO-ANN	0.8827	0.0094	0.8942	0.8750	0.8525	0.6459	0.0197	0.6721	0.6230	0.5583	0.7548	0.0387	0.8065	0.7097	0.7200
SCA-ANN	0.8769	0.0165	0.8942	0.8462	0.8525	0.6590	0.0123	0.6721	0.6393	0.5642	0.7548	0.0387	0.7742	0.6774	0.7429
SSA-ANN	0.8808	0.0077	0.8942	0.8750	0.8525	0.6393	0.0207	0.6557	0.6066	0.5440	0.7548	0.0329	0.8065	0.7097	0.7200
TLBO-ANN	0.8654	0.0228	0.8942	0.8269	0.8525	0.6492	0.0131	0.6557	0.6230	0.5519	0.7290	0.0329	0.7742	0.6774	0.7429
WOA-ANN	0.8827	0.0038	0.8846	0.8750	0.8305	0.6393	0.0147	0.6557	0.6230	0.5421	0.7871	0.0387	0.8065	0.7097	0.8065
**IPA-ANN ranking**			2		3			2		3			1		1

**Table 5:** Algorithms’ DS7–DS9 test accuracy and F1-score values for a single fold.

Algorithm	DS7					DS8					DS9				
	Mean	Std	Best	Worst	F1-Score	Mean	Std	Best	Worst	F1-Score	Mean	Std	Best	Worst	F1-Score
ABC-ANN	0.9333	0.0365	0.9667	0.8667	0.9666	0.6469	0.0224	0.6823	0.6198	0.6335	0.9080	0.0103	0.9195	0.8966	0.9197
BA-ANN	0.6467	0.0267	0.7000	0.6333	0.6861	0.6312	0.0083	0.6458	0.6198	0.5300	0.8000	0.0287	0.8276	0.7471	0.8212
BBO-ANN	0.9200	0.0163	0.9333	0.9000	0.9328	0.6625	0.0313	0.7083	0.6250	0.6777	0.9264	0.0172	0.9425	0.8966	0.9427
DE-ANN	0.8800	0.0452	0.9333	0.8000	0.9328	0.6906	0.0176	0.7083	0.6615	0.6804	0.9333	0.0046	0.9425	0.9310	0.9427
FA-ANN	0.8400	0.0533	0.9000	0.7667	0.8998	0.6625	0.0241	0.7083	0.6406	0.6653	0.8966	0.0163	0.9195	0.8736	0.9197
GA-ANN	0.9267	0.0827	**1.0000**	0.7667	**1.0000**	0.6458	0.0386	0.7135	0.5990	0.6821	0.8943	0.0285	0.9195	0.8391	0.9197
GBO-ANN	0.9267	0.0249	0.9667	0.9000	0.9666	0.6698	0.0112	0.6875	0.6562	0.6516	0.9218	0.0152	0.9425	0.8966	0.9424
GOA-ANN	0.6333	0.0000	0.6333	0.6333	0.4927	0.6365	0.0083	0.6510	0.6302	0.5673	0.8713	0.0366	0.9310	0.8161	0.9301
GWO-ANN	0.9133	0.0267	0.9333	0.8667	0.9328	0.7031	0.0174	0.7344	0.6823	0.6967	0.9310	0.0000	0.9310	0.9310	0.9310
HHO-ANN	0.8067	0.1143	0.9000	0.6000	0.8981	0.6792	0.0426	**0.7448**	0.6302	**0.7257**	0.8943	0.0169	0.9195	0.8736	0.9193
ICA-ANN	0.8533	0.1067	0.9667	0.7000	0.9666	0.6458	0.0189	0.6719	0.6146	0.5749	0.8897	0.0225	0.9195	0.8506	0.9182
IPA_1_2-ANN	0.7533	0.1408	0.9667	0.6000	0.9666	0.6417	0.0404	0.7135	0.5885	0.6821	0.8943	0.0285	0.9195	0.8391	0.9193
IPA_2_2-ANN	0.9200	0.0686	**1.0000**	0.8000	**1.0000**	0.6465	0.0201	0.6719	0.6146	0.5931	0.9080	0.0252	0.9425	0.8736	0.9427
IPA_3_3-ANN	0.9667	0.0516	**1.0000**	0.8667	**1.0000**	0.6594	0.0063	0.6667	0.6510	0.5948	0.9149	0.0200	0.9425	0.8851	0.9427
IPA_4_4-ANN	0.8267	0.0389	0.8667	0.7667	0.8631	0.6365	0.0141	0.6562	0.6146	0.5766	0.9172	0.0134	0.9310	0.8966	0.9310
MFO-ANN	0.7267	0.1306	0.9667	0.6333	0.9666	0.6458	0.0066	0.6510	0.6354	0.5673	0.8920	0.0156	0.9195	0.8736	0.9182
MRFO-ANN	0.9067	0.0249	0.9333	0.8667	0.9333	0.6604	0.0156	0.6823	0.6406	0.6442	0.9264	0.0156	**0.9540**	0.9080	**0.9538**
PSO-ANN	0.9133	0.0163	0.9333	0.9000	0.9328	0.6594	0.0273	0.7031	0.6302	0.6675	0.9172	0.0134	0.9310	0.8966	0.9314
SCA-ANN	0.9000	0.0211	0.9333	0.8667	0.9333	0.6646	0.0142	0.6875	0.6458	0.6249	0.9218	0.0086	0.9310	0.9080	0.9310
SSA-ANN	0.8867	0.0618	0.9667	0.8000	0.9666	0.6729	0.0217	0.7083	0.6510	0.6748	0.9011	0.0156	0.9195	0.8736	0.9201
TLBO-ANN	0.9200	0.0400	0.9667	0.8667	0.9666	0.6656	0.0250	0.7031	0.6302	0.6538	0.9195	0.0103	0.9310	0.9080	0.9310
WOA-ANN	0.7733	0.1218	0.9333	0.6333	0.9333	0.6323	0.0215	0.6562	0.6042	0.5512	0.8713	0.0401	0.9080	0.7931	0.9080
**IPA-ANN ranking**			1		1			3		3			2		2

**Table 6:** Test accuracy of conventional gradient-based ANN training baselines on the DS2 (Chess) and DS7 (Iris) datasets.

Optimizer	DS2 (Chess)				DS7 (Iris)			
	Mean	Std	Best	Worst	Mean	Std	Best	Worst
SGD	0.9892	0.0036	0.9938	0.9844	0.9667	0.0471	1.0000	0.8667
SGD-Momentum	0.9920	0.0015	0.9938	0.9891	0.9467	0.0163	0.9667	0.9333
RMSprop	0.9900	0.0042	0.9969	0.9828	0.9667	0.0258	1.0000	0.9333
Adam	0.9903	0.0033	0.9953	0.9844	0.9600	0.0291	1.0000	0.9333
Adagrad	0.9825	0.0069	0.9922	0.9734	0.9700	0.0277	1.0000	0.9333

**Table 7:** Average rank of each of the 22 algorithm configurations across all nine datasets (DS1–DS9), computed from the mean test accuracy values reported in Table 3, Table 4 and Table 5 (rank 1 = best per dataset). The Friedman test yielded $\chi^{2}=44.279$ (df=21, p=0.0022), rejecting the null hypothesis of equal performance among all compared algorithms across the nine datasets at α=0.05.

Rank	Algorithm	Average Rank
1	GWO-ANN	5.000
2	DE-ANN	6.167
3	IPA_3_3-ANN	7.778
4	MRFO-ANN	9.500
5	TLBO-ANN	9.556
6	SCA-ANN	9.556
7	PSO-ANN	9.556
8	GBO-ANN	9.778
9	BBO-ANN	10.222
10	ABC-ANN	10.778
11	GA-ANN	11.000
12	IPA_2_2-ANN	11.111
13	SSA-ANN	12.611
14	HHO-ANN	12.889
15	IPA_4_4-ANN	12.944
16	MFO-ANN	13.278
17	FA-ANN	13.722
18	IPA_1_2-ANN	13.778
19	WOA-ANN	15.111
20	GOA-ANN	15.444
21	ICA-ANN	16.167
22	BA-ANN	17.056

**Table 8:** Pairwise Wilcoxon signed-rank test results (with Holm–Bonferroni correction across 21 simultaneous comparisons) between IPA_3_3-ANN and each of the remaining 21 algorithms, using the paired mean test accuracy values across all nine datasets (DS1–DS9) as the paired observations (n=9).

Compared Method	*p*-Value	Holm-Adjusted *p*	Significant	Winner (By Mean)
WOA-ANN	0.0273	0.5742	No	IPA_3_3-ANN
BA-ANN	0.0391	0.7812	No	IPA_3_3-ANN
IPA_1_2-ANN	0.0391	0.7812	No	IPA_3_3-ANN
GOA-ANN	0.0391	0.7812	No	IPA_3_3-ANN
ICA-ANN	0.0547	0.9297	No	IPA_3_3-ANN
IPA_2_2-ANN	0.0742	1.0000	No	IPA_3_3-ANN
FA-ANN	0.0742	1.0000	No	IPA_3_3-ANN
MFO-ANN	0.0742	1.0000	No	IPA_3_3-ANN
GA-ANN	0.0977	1.0000	No	IPA_3_3-ANN
ABC-ANN	0.0977	1.0000	No	IPA_3_3-ANN
IPA_4_4-ANN	0.1484	1.0000	No	IPA_3_3-ANN
HHO-ANN	0.2500	1.0000	No	IPA_3_3-ANN
SSA-ANN	0.2500	1.0000	No	IPA_3_3-ANN
MRFO-ANN	0.4258	1.0000	No	IPA_3_3-ANN
BBO-ANN	0.5469	1.0000	No	IPA_3_3-ANN
GBO-ANN	0.5703	1.0000	No	IPA_3_3-ANN
TLBO-ANN	0.5703	1.0000	No	IPA_3_3-ANN
SCA-ANN	0.5703	1.0000	No	IPA_3_3-ANN
PSO-ANN	0.5781	1.0000	No	IPA_3_3-ANN
GWO-ANN	0.8203	1.0000	No	GWO-ANN
DE-ANN	1.0000	1.0000	No	IPA_3_3-ANN

**Table 9:** Descriptive statistics of test accuracy obtained via repeated stratified 5-fold cross-validation (5 repetitions, 25 folds in total) for the DS6 dataset.

Algorithm	Mean	Std	Median	Q1	Q3	Min	Max	IQR
ABC-ANN	0.8219	0.0243	0.8289	0.7975	0.8451	0.7870	0.8451	0.0476
BA-ANN	0.7948	0.0114	0.7870	0.7870	0.8107	0.7870	0.8129	0.0237
BBO-ANN	0.8374	0.0225	0.8425	0.8129	0.8632	0.8129	0.8645	0.0503
DE-ANN	0.8129	0.0045	0.8133	0.8095	0.8166	0.8064	0.8193	0.0071
FA-ANN	0.8219	0.0207	0.8295	0.7973	0.8390	0.7935	0.8451	0.0417
GA-ANN	0.8077	0.0084	0.8067	0.8000	0.8175	0.8000	0.8193	0.0175
GBO-ANN	0.8310	0.0194	0.8129	0.8129	0.8516	0.8129	0.8516	0.0387
GOA-ANN	0.7950	0.0113	0.7870	0.7870	0.8058	0.7870	0.8129	0.0188
GWO-ANN	0.8287	0.0120	0.8193	0.8193	0.8451	0.8193	0.8451	0.0258
HHO-ANN	0.8232	0.0180	0.8303	0.8111	0.8387	0.7935	0.8387	0.0276
ICA-ANN	0.8232	0.0161	0.8129	0.8129	0.8377	0.8129	0.8516	0.0248
IPA_1_2-ANN	0.8167	0.0168	0.8138	0.8008	0.8287	0.8000	0.8451	0.0279
IPA_2_2-ANN	0.8090	0.0032	0.8064	0.8064	0.8129	0.8064	0.8129	0.0065
IPA_3_3-ANN	0.8129	0.0164	0.8153	0.7935	0.8278	0.7935	0.8387	0.0343
IPA_4_4-ANN	0.8154	0.0196	0.8000	0.8000	0.8360	0.8000	0.8451	0.0360
MFO-ANN	0.8025	0.0148	0.8011	0.7870	0.8161	0.7870	0.8258	0.0291
MRFO-ANN	0.8245	0.0154	0.8254	0.8079	0.8414	0.8064	0.8451	0.0335
PSO-ANN	0.8374	0.0327	0.8340	0.8064	0.8701	0.8064	0.8903	0.0637
SCA-ANN	0.8154	0.0141	0.8156	0.8030	0.8316	0.7935	0.8322	0.0286
SSA-ANN	0.8219	0.0125	0.8205	0.8107	0.8348	0.8064	0.8387	0.0241
TLBO-ANN	0.8237	0.0065	0.8239	0.8156	0.8304	0.8156	0.8323	0.0148
WOA-ANN	0.8154	0.0252	0.7982	0.7935	0.8347	0.7935	0.8580	0.0412

**Table 10:** Pairwise Wilcoxon signed-rank test results (with Holm–Bonferroni correction) between IPA_1_2-ANN and the remaining 21 algorithms on the DS6 dataset.

Compared Method	*p*-Value	Holm-Adjusted *p*	Significant	Winner
ABC-ANN	0.4470	1.0000	No	ABC-ANN
BA-ANN	0.0001	0.0019	Yes	IPA_1_2-ANN
BBO-ANN	0.0002	0.0041	Yes	BBO-ANN
DE-ANN	0.3819	1.0000	No	IPA_1_2-ANN
FA-ANN	0.3173	1.0000	No	FA-ANN
GA-ANN	0.0425	0.5944	No	IPA_1_2-ANN
GBO-ANN	0.0080	0.1284	No	GBO-ANN
GOA-ANN	0.0001	0.0025	Yes	IPA_1_2-ANN
GWO-ANN	0.0068	0.1152	No	GWO-ANN
HHO-ANN	0.2310	1.0000	No	HHO-ANN
ICA-ANN	0.1283	1.0000	No	ICA-ANN
IPA_2_2-ANN	0.0977	1.0000	No	IPA_1_2-ANN
IPA_3_3-ANN	0.2758	1.0000	No	IPA_1_2-ANN
IPA_4_4-ANN	0.7943	0.7943	No	IPA_1_2-ANN
MFO-ANN	0.0063	0.1136	No	IPA_1_2-ANN
MRFO-ANN	0.1096	1.0000	No	MRFO-ANN
PSO-ANN	0.0185	0.2782	No	PSO-ANN
SCA-ANN	0.7570	1.0000	No	IPA_1_2-ANN
SSA-ANN	0.2584	1.0000	No	SSA-ANN
TLBO-ANN	0.0875	1.0000	No	TLBO-ANN
WOA-ANN	0.7366	1.0000	No	IPA_1_2-ANN

Summary: Proposed Method = IPA_1_2-ANN; Wins = 10; Ties = 0; Losses = 11.

**Table 11:** Descriptive statistics of test accuracy obtained via repeated stratified 5-fold cross-validation (5 repetitions, 25 folds in total) for the DS7 dataset.

Algorithm	Mean	Std	Median	Q1	Q3	Min	Max	IQR
ABC-ANN	0.9466	0.0194	0.9600	0.9290	0.9600	0.9133	0.9600	0.0310
BA-ANN	0.7081	0.1361	0.6333	0.6333	0.7007	0.6333	0.9533	0.0674
BBO-ANN	0.9746	0.0152	0.9745	0.9600	0.9886	0.9600	1.0000	0.0286
DE-ANN	0.9666	0.0124	0.9694	0.9576	0.9797	0.9466	0.9800	0.0221
FA-ANN	0.9386	0.0276	0.9294	0.9133	0.9616	0.9133	0.9800	0.0483
GA-ANN	0.8999	0.0851	0.9600	0.8341	0.9600	0.7533	0.9600	0.1259
GBO-ANN	0.9854	0.0133	0.9733	0.9733	1.0000	0.9733	1.0000	0.0267
GOA-ANN	0.5683	0.1550	0.6533	0.5806	0.6533	0.2866	0.6533	0.0727
GWO-ANN	0.9143	0.1560	1.0000	0.9227	1.0000	0.6333	1.0000	0.0773
HHO-ANN	0.9560	0.0554	0.9848	0.9260	1.0000	0.8600	1.0000	0.0740
ICA-ANN	0.9360	0.0848	0.9884	0.8492	1.0000	0.7933	1.0000	0.1508
IPA_1_2-ANN	0.8440	0.0816	0.7840	0.7800	0.9253	0.7800	0.9733	0.1453
IPA_2_2-ANN	0.9692	0.0165	0.9800	0.9564	0.9800	0.9400	0.9800	0.0237
IPA_3_3-ANN	0.9318	0.0635	0.9733	0.8571	0.9733	0.8200	0.9733	0.1162
IPA_4_4-ANN	0.9546	0.0595	1.0000	0.9006	1.0000	0.8533	1.0000	0.0994
MFO-ANN	0.8944	0.1430	1.0000	0.7415	1.0000	0.6533	1.0000	0.2585
MRFO-ANN	0.9840	0.0129	0.9859	0.9717	0.9984	0.9667	1.0000	0.0267
PSO-ANN	0.9813	0.0159	0.9853	0.9630	0.9992	0.9600	1.0000	0.0363
SCA-ANN	0.9772	0.0033	0.9800	0.9733	0.9800	0.9733	0.9800	0.0067
SSA-ANN	0.9733	0.0290	0.9846	0.9498	1.0000	0.9266	1.0000	0.0502
TLBO-ANN	0.9720	0.0144	0.9712	0.9572	0.9833	0.9533	0.9933	0.0261
WOA-ANN	0.7600	0.1403	0.6707	0.6333	0.8769	0.6333	0.9866	0.2436

**Table 12:** Pairwise Wilcoxon signed-rank test results (with Holm–Bonferroni correction) between IPA_2_2-ANN and the remaining 21 algorithms on the DS7 dataset.

Compared Method	*p*-Value	Holm-Adjusted *p*	Significant	Winner
ABC-ANN	0.0005	0.0086	Yes	IPA_2_2-ANN
BA-ANN	<0.0001	0.0002	Yes	IPA_2_2-ANN
BBO-ANN	0.3081	1.0000	No	BBO-ANN
DE-ANN	0.4140	1.0000	No	IPA_2_2-ANN
FA-ANN	0.0006	0.0088	Yes	IPA_2_2-ANN
GA-ANN	0.0002	0.0032	Yes	IPA_2_2-ANN
GBO-ANN	0.0021	0.0274	Yes	GBO-ANN
GOA-ANN	<0.0001	0.0002	Yes	IPA_2_2-ANN
GWO-ANN	0.4515	1.0000	No	IPA_2_2-ANN
HHO-ANN	0.8609	0.8609	No	IPA_2_2-ANN
ICA-ANN	0.4414	1.0000	No	IPA_2_2-ANN
IPA_1_2-ANN	0.0000	0.0008	Yes	IPA_2_2-ANN
IPA_3_3-ANN	0.0024	0.0284	Yes	IPA_2_2-ANN
IPA_4_4-ANN	0.5426	1.0000	No	IPA_2_2-ANN
MFO-ANN	0.2502	1.0000	No	IPA_2_2-ANN
MRFO-ANN	0.0009	0.0123	Yes	MRFO-ANN
PSO-ANN	0.0100	0.1099	No	PSO-ANN
SCA-ANN	0.1000	1.0000	No	SCA-ANN
SSA-ANN	0.4413	1.0000	No	SSA-ANN
TLBO-ANN	0.3962	1.0000	No	TLBO-ANN
WOA-ANN	0.0000	0.0008	Yes	IPA_2_2-ANN

Summary: Proposed Method = IPA_2_2-ANN; Wins = 14; Ties = 0; Losses = 7.

**Table 13:** Mean per-evaluation CPU time and total CPU time over 10,000 evaluations for the compared algorithms on the DS7 dataset, sorted in ascending order of total execution time.

Algorithm	Mean Time/Evaluation (s)	Std (s)	Total Time (s)	Total Time (min)
PSO-ANN	0.3103	0.0748	3103.07	51.72
WOA-ANN	0.3118	0.0761	3118.18	51.97
MRFO-ANN	0.3125	0.0754	3125.19	52.09
IPA_3_3-ANN	0.3130	0.0748	3129.53	52.16
IPA_2_2-ANN	0.3130	0.0787	3130.39	52.17
MFO-ANN	0.3142	0.0775	3142.44	52.37
ICA-ANN	0.3145	0.0764	3144.92	52.42
BA-ANN	0.3151	0.0770	3151.45	52.52
IPA_1_2-ANN	0.3152	0.0778	3152.07	52.53
IPA_4_4-ANN	0.3155	0.0768	3154.70	52.58
TLBO-ANN	0.3157	0.0791	3156.89	52.61
GWO-ANN	0.3165	0.0734	3164.83	52.75
ABC-ANN	0.3173	0.0767	3172.89	52.88
HHO-ANN	0.3174	0.0793	3174.41	52.91
SSA-ANN	0.3178	0.0791	3178.06	52.97
GA-ANN	0.3180	0.0766	3180.01	53.00
SCA-ANN	0.3186	0.0763	3186.17	53.10
DE-ANN	0.3187	0.0778	3187.29	53.12
GBO-ANN	0.3195	0.0804	3195.06	53.25
BBO-ANN	0.3196	0.0798	3196.21	53.27
GOA-ANN	0.3212	0.0777	3212.14	53.54
FA-ANN	0.3664	0.2608	3664.08	61.07

## Data Availability

The original contributions presented in this study are included in the article. Further inquiries can be directed to the corresponding author.
